# Integration of genetic and genomics resources in einkorn wheat enables precision mapping of important traits

**DOI:** 10.1038/s42003-023-05189-z

**Published:** 2023-08-12

**Authors:** Gautam Saripalli, Laxman Adhikari, Cameron Amos, Ashraf Kibriya, Hanin Ibrahim Ahmed, Matthias Heuberger, John Raupp, Naveenkumar Athiyannan, Thomas Wicker, Michael Abrouk, Sydney Wallace, Seyedali Hosseinirad, Parveen Chhuneja, Janelle Livesay, Nidhi Rawat, Simon G. Krattinger, Jesse Poland, Vijay Tiwari

**Affiliations:** 1 Department of Plant Science and Landscape Architecture, University of Maryland, College Park, MD 20783 USA; 2 Plant Science Program, Biological and Environmental Science and Engineering Division, King Abdullah University of Science and Technology (KAUST), Thuwal, 23955-6900 Saudi Arabia; 3 Center for Desert Agriculture, King Abdullah University of Science and Technology, Thuwal, Saudi Arabia; 4 Department of Plant Pathology, Kansas State University, Manhattan, KS 66506 USA; 5 Department of Plant and Microbial Biology, University of Zurich, Zurich, Switzerland; 6 Wheat Genetics Resource Center and Department of Plant Pathology, Kansas State University, Manhattan, KS 66506 USA; 7 School of Agricultural Biotechnology, Punjab Agricultural University, Ludhiana, 141004 Punjab India

**Keywords:** Genetics, Quantitative trait, Comparative genomics

## Abstract

Einkorn wheat (*Triticum monococcum*) is an ancient grain crop and a close relative of the diploid progenitor (*T. urartu*) of polyploid wheat. It is the only diploid wheat species having both domesticated and wild forms and therefore provides an excellent system to identify domestication genes and genes for traits of interest to utilize in wheat improvement. Here, we leverage genomic advancements for einkorn wheat using an einkorn reference genome assembly combined with skim-sequencing of a large genetic population of 812 recombinant inbred lines (RILs) developed from a cross between a wild and a domesticated *T. monococcum* accession. We identify 15,919 crossover breakpoints delimited to a median and average interval of 114 Kbp and 219 Kbp, respectively. This high-resolution mapping resource enables us to perform fine-scale mapping of one qualitative (red coleoptile) and one quantitative (spikelet number per spike) trait, resulting in the identification of small physical intervals (400 Kb to 700 Kb) with a limited number of candidate genes. Furthermore, an important domestication locus for brittle rachis is also identified on chromosome 7A. This resource presents an exciting route to perform trait discovery in diploid wheat for agronomically important traits and their further deployment in einkorn as well as tetraploid pasta wheat and hexaploid bread wheat cultivars.

## Abstract (web summary)

Integration of skim sequencing data for a recombinant inbred line population derived from a cross between wild and domesticated einkorn wheat accessions with a reference genome assembly enables high-resolution mapping of agronomic traits.

## Introduction

Being a staple source of calories for around 40% of the human population, common wheat (hexaploid bread wheat; 2n = 6x = 42; *Triticum aestivum* L.) is a critical crop for global food security^1^. Global wheat production faces continuous threats from changing climatic conditions including new abiotic stresses and rapidly evolving pests and diseases^2^. Genetic improvement of crop plants is the most sustainable agricultural approach for meeting these challenges but requires a continuous process of identification, characterization, and deployment of useful allelic variants for agronomically important genes in breeding programs^3–7^.

Diploid A-genome einkorn wheat (*Triticum monococcum* L. subsp. *monococcum* (2n = 2x = 14, A^m^A^m^ genome), is a model plant for wheat and other Triticeae species. *T. monococcum* is one of the first domesticated and oldest cultivated crops, with a history dating back about 12,000 years^8,9^. Being an agricultural founder crop with a long history of cultivation in various geographical and environmental regions, einkorn wheat is an important source of genes for improving modern wheat for resistance against biotic and abiotic stresses^10–19^. The einkorn wheat holds impressive nutritional content and high genetic polymorphism, and its vast genetic potential for wheat improvement has sparked renewed interest in this ancient crop.

With a close relationship to polyploid wheat and simplified genetics as a diploid species, einkorn is a practical model for the functional genetics of wheat. Comparative genomics analysis between the A-genomes of bread wheat and *T. monococcum* revealed high sequence similarity, gene structure conservation, and very limited gene loss and chromosomal rearrangement between the two genomes^20–23^. Several useful resources, namely: association panels^9,24^, genetic populations^25–27^, TILLING population^28^, BAC libraries^29^, and high-quality reference genome assembly^30^ have been established for *T. monococcum*, highlighting its suitability as a model for wheat functional genomics.

Einkorn wheat also offers a unique opportunity to study domestication and selection-related evolutionary history as it is the only diploid wheat species for which there are both wild and cultivated types^9,17,31^. Because of the contrasting characters of wild and cultivated einkorn^32^, mapping populations have been developed from the crosses between wild and cultivated einkorn accessions to understand the genetic architecture of domestication and agronomic traits in einkorn wheat^11,33–35^.

Quantitative trait locus (QTL) mapping in einkorn wheat has identified key loci for some important traits which include domestication traits such as brittle rachis, agronomic traits like spikelet number per spike, heading date and grain number per spikelet, and biotic stresses like powdery mildew, stripe rust and nematode resistance^11,25,33,34,36–39^. However, since these studies were mostly based on smaller populations and were characterized using low-density genetic maps, mapping resolution of the genetic and trait mapping did not allow precise identification of the QTL regions. This study overcomes the population size and marker density limitations by using a larger panel of recombinant inbred lines (RILs) and thousands of loci.

Recently, we developed high-quality reference assemblies for a wild and a domesticated einkorn accession^30^ along with a sequence-indexed panel of diverse einkorn accessions. To fully utilize these genomic resources and uncover the genetic potential of einkorn wheat, molecular breeding strategies must be implemented including the genetic mapping of important agronomic traits.

In this study, we integrate genetic and genomic resources including reference genome assemblies and unique germplasm sets incorporating wild and domesticated einkorn wheat to understand the recombination patterns and their distributions across the A-genome and demonstrate the application of these integrated resources in fine-scale analysis of agronomically important genes to improve diploid and polyploid wheat cultivars. The major traits that we targeted in this study include qualitative traits such as coleoptile color or red coleoptile (Rc) and blue aleurone (Ba), quantitative agronomic traits like plant height (PH), spikelet number per spike (SPLSPK), spike length (SpkLng) and spikes per plant (SPP) and a key domestication trait; brittle rachis (Btr). We also fine-mapped two important QTL for SPLSPK and Rc. Each of these traits has a specific importance as follows: (i) Coleoptile color or red coleoptile is an important morphological trait that is mainly due to the accumulation of anthocyanins. The red coleoptile also protects the emerging shoots from different abiotic stresses like strong sunlight, drought, and cold^40,41^. (ii) Spikelet number per spike is an important agronomic trait that is the major determinant of the overall grain yield of the plant. (iii) Brittle rachis is a key domestication trait that leads to the divergence of wild and domesticated einkorn wheat. While wild einkorn wheat forms have a fragile rachis, which promotes seed dispersal, the latter’s domesticated forms of wheat have a rachis that is non-fragile and breaks only when vigorously threshed, a prominent component of the domestication syndrome^42^.

Further, we also conducted the marker trait association (MTA) analysis using the same RIL panel, but with multiple folds of markers (1.6 M SNPs) achieved through the imputation of whole-genome variants identified in the two parents. Here we showed that low-cost skim-sequencing can be effectively applied to large mapping population genotyping and precise trait mapping with higher resolution. Additionally, we present a bioinformatics pipeline that can be employed to genotype a breeding population using low-coverage sequencing data.

## Results

### Genotyping data for parents (L95 and L96) and RIL population

We utilized a RIL population consisting of 812 lines derived from a cross between wild einkorn (*T. monococcum* spp. *aegilopoides*; accession TA291, syn. TA4342-L95, subsequently referred to L95) and a domesticated einkorn (*T. monococcum* spp. *monococcum*, accession TA10868, syn. TA4342-L96, subsequently referred to L96) accession. This population was originally described by Singh et al. (2007)^25^ and was expanded to 812 RILs. To identify variants for genotyping with skim-sequencing in the RIL population, we first generated whole-genome sequencing (WGS) data of the two RIL parents (L95 and L96) corresponding to approximately 9.1x coverage of the 5.1 Gb einkorn genome. To genotype the RIL population efficiently and cost-effectively, we utilized skim-sequencing by targeting the population to an average depth of 0.03 to 0.2x coverage^43^. For genotyping, we first discovered variants for two parents L95 and L96 by aligning the WGS data to the recently completed einkorn genome assemblies^30^. We observed 90% overall alignment for L95 and 85% overall alignment for L96 when aligned to the TA299 (wild einkorn) reference assembly. Similarly, with mapping to the TA10622 (domesticated einkorn) assembly, L95 parent had about 85% overall alignment and L96 had 92% overall read alignment. Therefore, these mapping stats were harmonized for the subspecies types of these einkorn RIL parents, L95 (wild) and L96 (domesticated), and the assemblies TA299 (wild) and TA10622 (domesticated) used for mapping. The five replicates of each parent sequenced with the skim-sequencing RILs also confirmed the precision of genotyping. In this study, for genetic linkage and trait mapping, we used only genotyping information generated with read mapping on the TA299 assembly^30^.

### Variant calling in parents and RILs

**Fig. 1:**
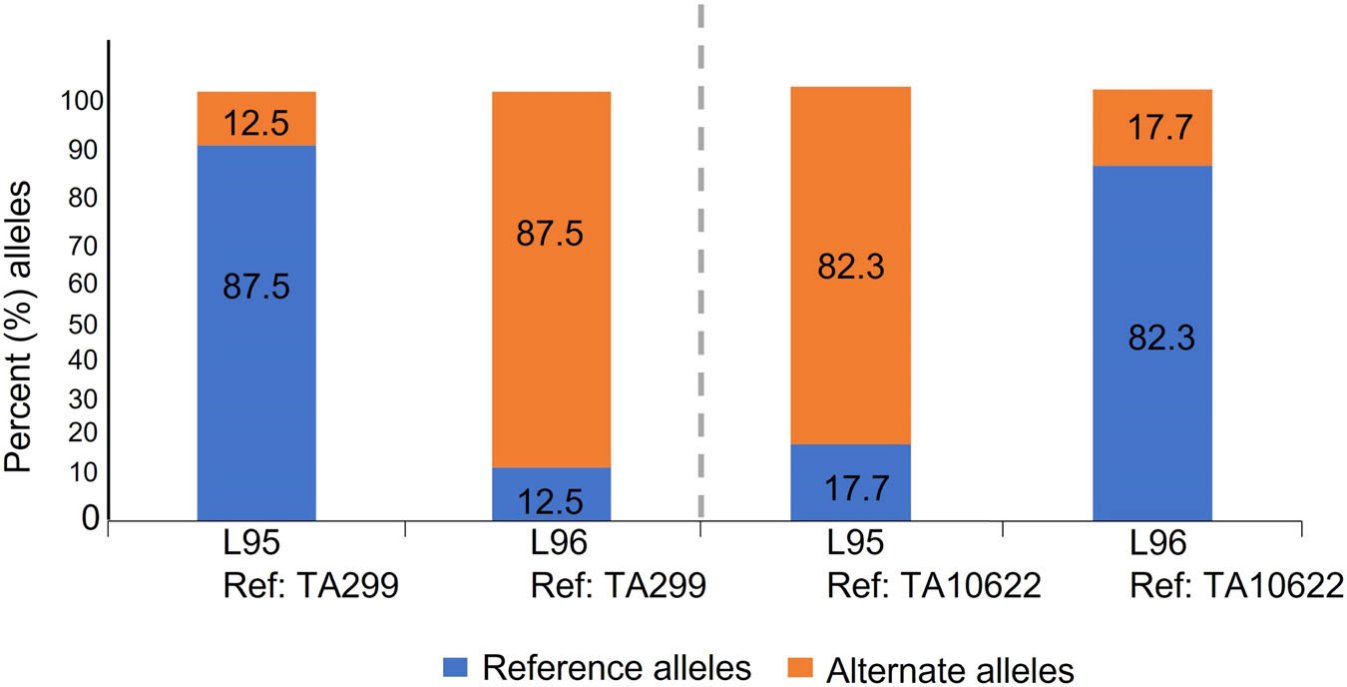
The proportion of reference and alternate alleles called for L95 (wild) and L96 (cultivated) *T. monococcum* when the variants were called on TA299 (wild) and TA10622 (domesticated) reference genome assemblies. For each of the respective assemblies, the proportion of reference alleles are shown in blue and alternate alleles in orange. The numbers inside the bars indicate the exact percentage of reference and alternate alleles when called on TA299 and TA10622.

**Fig. 2:**
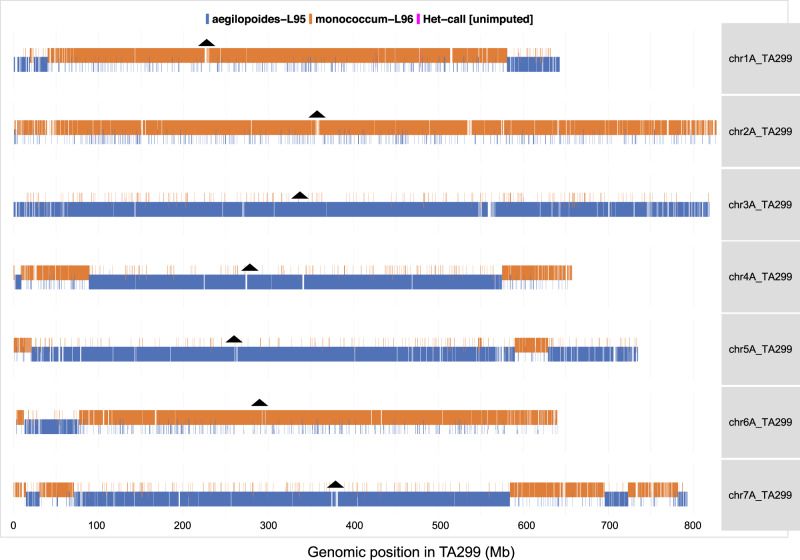
A recombinant inbred line (2013-31-153) showing distribution of wild (L95) and cultivated (L96) parental alleles. The orange bars are individual variant calls matching parent L96, in blue for matching parent L95 and a few magenta bars represent the heterozygous. The black triangle displays the centromeric positions in each of the TA299 chromosomes.

When aligning to the TA299 genome, we observed 87.5% reference (ref) alleles and 12.5% alternate (alt) alleles for parent L95, commensurate with this parent being a wild einkorn similar to TA299. It was the reverse situation for domesticated parent L96 (12.5 % ref allele and 87.5 % alt allele on TA299 genome) (Fig. 1). We initially performed a quality filter for these variants as described^43^. Briefly, each site was filtered based on read and allele depths so that we kept SNPs with minimum and maximum read depths of ≥6 and ≤100, respectively, and reference and alternate allele read depths of ≥3. The filtered SNPs list included only the segregating loci which are homozygous and non-missing genotypes of the two parents. We identified 16.6 M (16,687,336) total filtered variants in two parents, from which we called the same sites on the skim-seq RIL population^43^. After identifying the variants on RILs, we assigned each SNP allele call to the parental allele by matching the genotype of individual RILs to the genotype of parents (L95 or L96) for the corresponding sites. These genotype calls were visualized for the progeny which enabled robust identification of genomic regions as P1 (L95 allele), P2 (L96 allele), and heterozygous (each allele from either parent) (Fig. 2). For the construction of a linkage map and QTL analysis, the consensus genotype was called within the 1-Mb non-overlapping windows based on the proportions of both the parents L95 (P1) and L96 (P2) using a threshold (≥0.7) within the windows. In this manner, a total of 5096 bins of 1 Mb size covered the entire genome of *T*. *monococcum* (TA299). Twelve highly heterozygous RILs were removed for further analysis of linkage and genetic mapping to maintain the genetic make-up of the inbred population.

From this genotyping approach, we obtained an average 264,825 typed variants per RIL, giving sufficient genotype coverage with >10,000 genotyped sites per chromosome to delimit long parental haplotypes with an expected two to five recombination breakpoints per chromosome. From this dataset, we then identified recombination breakpoints and constructed a genetic bin map (Fig. 1). The 16.6 million filtered variants identified in the datasets were evenly distributed to all seven chromosomes (Supplementary Fig. 1). The telomeric ends had a lower SNPs density than the pericentric areas, which indicated the telomeric regions are highly diverse and had a low mapping frequency. The number of observed variants on the genomic regions is influenced by the total number of reads mapped that can be reliably mapped to a given region which is a function of the divergence from the reference genome as well as based on the relative diversity within the population of study, as in this case between the wild and the domesticated einkorn parents. The distribution of either parental alleles (L95 and L96) or heterozygous in the RILs separated the genome segments parental source for individual RIL and genotyped them properly (Fig. 2). There were slightly more L95 alleles than L96 alleles in the entire population and a very low number of heterozygous regions.

### Recombination patterns and distribution across the einkorn genome

**Fig. 3:**
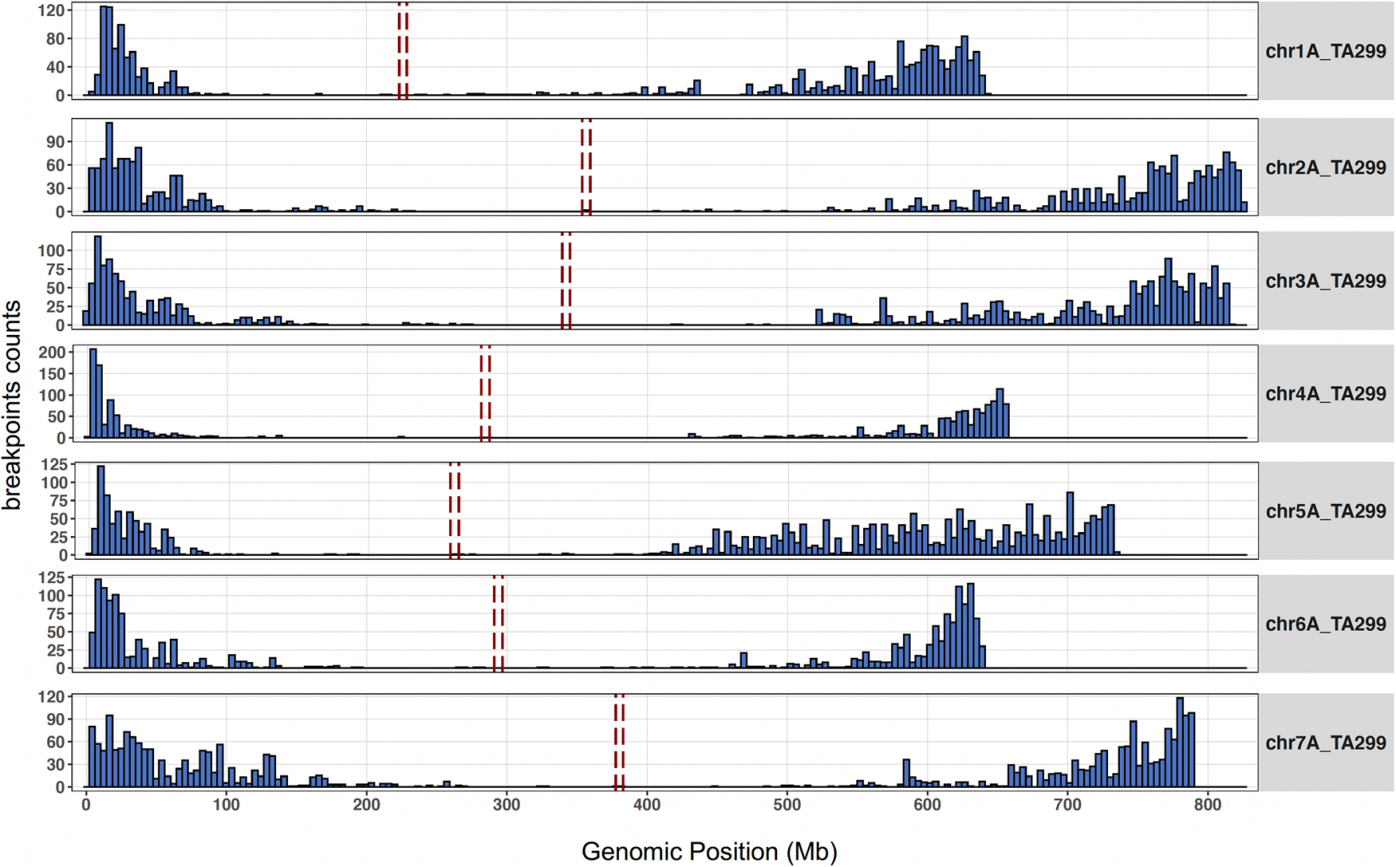
Distribution of genome-wide recombination breakpoints for each of the seven einkorn chromosomes. The number of observed recombination crossover sites observed in the population. The dashed red vertical lines show the centromeric regions in the TA299 genome.

Recombination breakpoints were identified between informative flanking markers using a newly developed roaming score, similar to the approach of Huang et al. (2009)^44^ but optimized for the low coverage data generated with skim-seq (Supplementary Note 1; Fig. 3 and Supplementary Fig. 2) Using this approach, we identified a total of 15,919 crossover breakpoints in the population. Even with very low sequencing coverage of 0.03x, we could delineate the crossover breakpoints accurately with a median interval length of 114 Kbp, and a mean interval of 219 Kbp (Fig. 3). Using reverse calculation of the genetic map length based on a RIL population size of 812 lines and 15,919 crossovers, we estimated the genetic map length at 1960 cM, consistent with the expected genetic map length of a RIL population for diploid wheat. As expected for Triticeae genomes, we observed an almost complete lack of recombination in the centromeric regions. This ‘recombination desert’ extended to approximately 50% of the center of the chromosomes with crossover breakpoints localized to the first 20% to 30% of each chromosome arm (Fig. 3).

### Imputation of genotyping data and genetic mapping of agronomically important traits

**Fig. 4:**
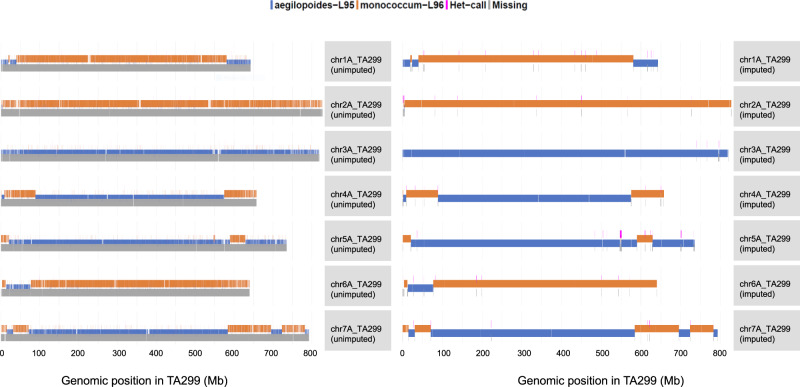
Genome-wide missing data before (left) and after imputation (right) for a recombinant inbred line (2013-31-153). The chromosomes after imputation (imputed) had almost no missing data as compared to before imputation (unimputed). After imputation, the missing data (gray bars) were replaced by either of the parental alleles.

Imputation of the genotype data using LB-impute^45^, enabled us to impute almost all the genotypes with only ~1.3% missing genotypes remaining after imputation, compared to the original 98.1% missing genotypes on average (Fig. 4). To test the imputation accuracy, we used the hold-out method, we randomly masked 3% of the non-missing genotypes independently on each of the seven chromosomes. Then, we calculated the imputation accuracy for each chromosome and each RIL followed by the computation of the average imputation accuracy for the entire dataset, which was found to be 95.5%. After imputation, about 16.4 M (98%) variants had allelic info for ~98% of individuals. The imputation algorithm also performed well in the heterozygous regions where it replaced intermixed P1 (L95) and P2 (L96) genotypes as heterozygous (H) (Fig. 3). The imputed data was used to run the MTA analysis as described below.

All the above-mentioned analysis including the imputation, identification of recombinant break points, etc. was performed on 812 RILs that were skim-sequenced. However, for identifying QTL using interval mapping, a subset of 635 RILs (out of 812) were used for which phenotypic data was available. Therefore, to maintain consistency with the lines used for phenotyping, a separate genetic linkage map was generated for QTL analysis using 635 RILs. This linkage map consisted of 1076 1-Mb binned  markers distributed over all seven chromosomes (8 linkage groups) with a total genetic distance of 480.37 cM (for details, see Supplementary Data 1; Supplementary Table 1; Supplementary Fig. 3 and Supplementary Note 2).

### Population phenotyping and trait mapping

**Fig. 5:**
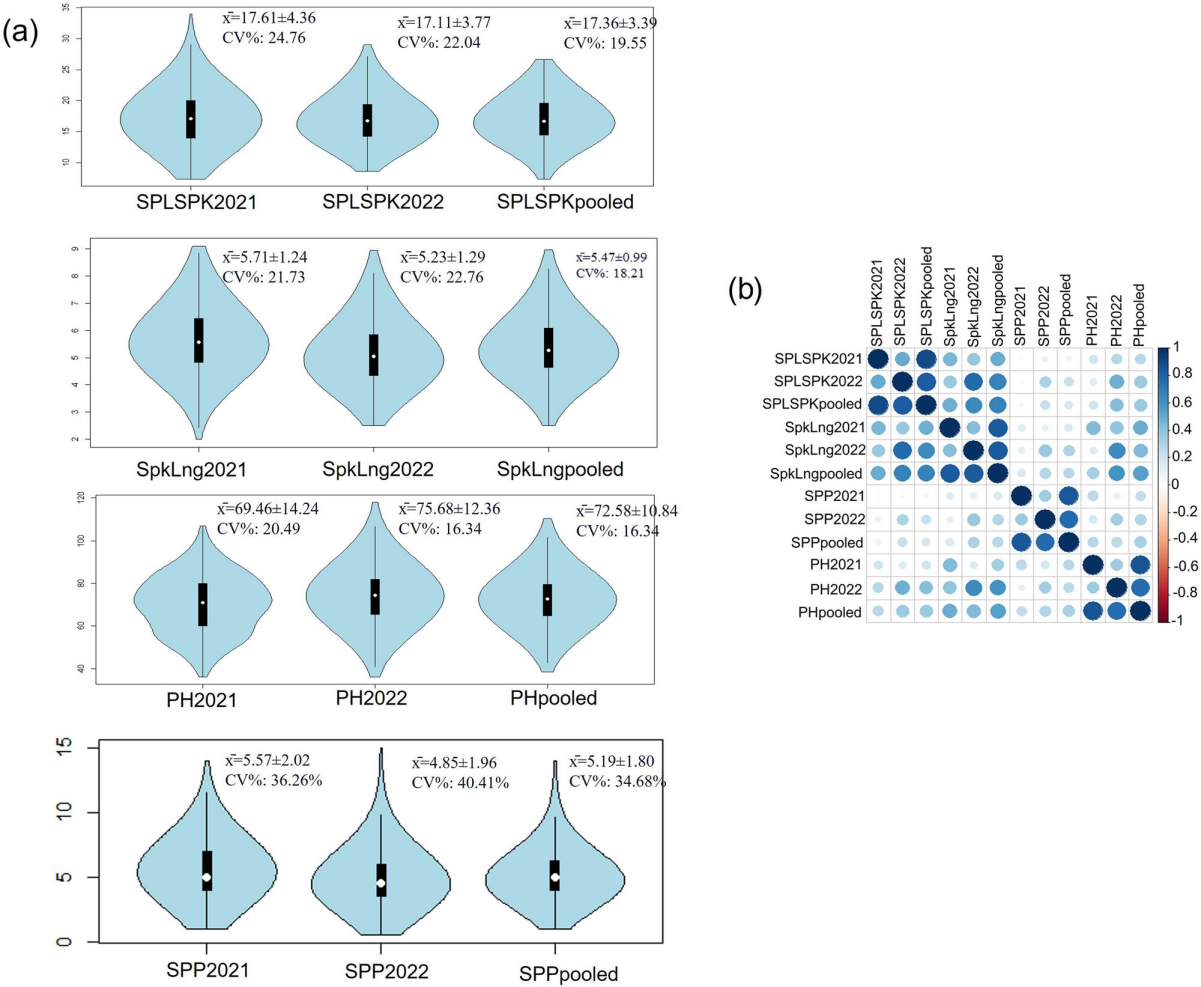
Frequency distribution of traits and correlation of traits. **a** Violin plots depicting the phenotypic distribution of yield related quantitative traits. Descriptive statistics for each trait including mean (x̄), coefficient of variation (CV%) and Pearson correlation coefficient (Corr) between the two-year data as well as each year with the pooled data. The white dot in the center of the plot represents the median and the length of the black shaded rectangular box indicates the interquartile range (**b**) Corrplot showing Pearson correlation coefficients across the years and within the yield related quantitative traits.

**Fig. 6:**
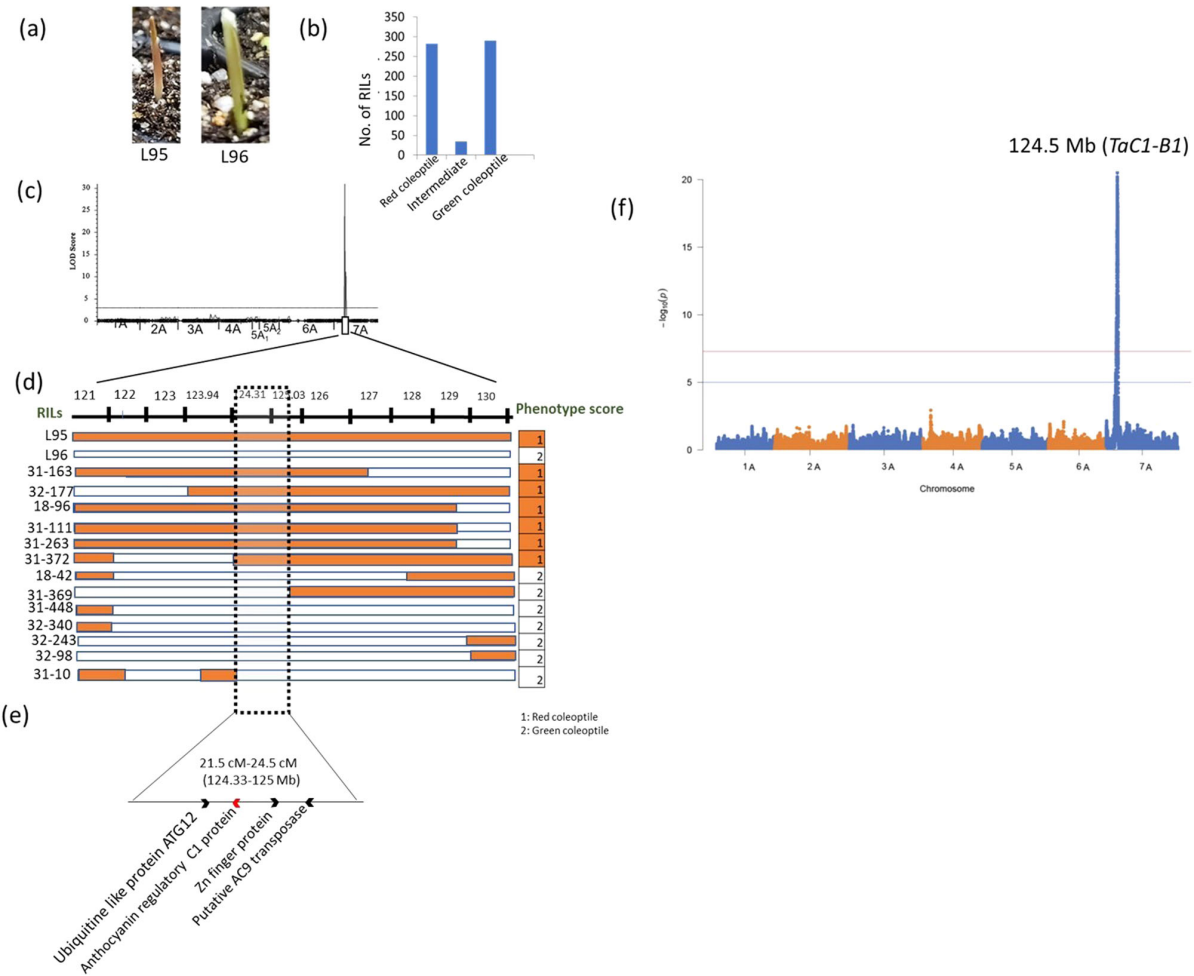
QTL and underlying candidate genes for coleoptile color (Rc). **a** Image depicting the phenotypic variation for Rc (**b**) distribution of RILs for Rc (**c**) QTL peak obtained using IciMapping software (**d**) recombinants identified in the RILs for Rc along with the physical interval; the text on the left of the horizontal bar indicates RILs codes and the values on the right in orange shade indicate the trait values for Rc; L95 and L96 are the two parents used for generating the RILs. The horizontal bars indicate the genomic region covered by the QTL in the interval 808–810 Mb; each bar indicates each recombinant inbred line (RIL). The orange shaded regions in the horizontal bars represent the genomic region for the parent 1 (i.e., TA4342-L95) whereas the white shaded region in the horizontal bars indicate the genomic region for the parent 2 (i.e., TA4342-L96). **e** Annotated genes identified in the delimited physical interval of 124.3 to 125 Mb and (**f**) results from association mapping analysis using all 1.6 M imputed variants obtained for Rc where the x-axis shows physical position of each variant site on the reference genome and the vertical axis showing the -log10 of the *p*-value from association test. The lower blue horizontal line refers to the Bonferroni-corrected genome-wide significance threshold & the upper red line indicates a highly stringent genome-wide significance threshold of *P*-value ≤ 5 × 10^−8^. The location of a candidate orthologous gene identified at 124.5 Mb corresponding to the strongest associated variant site.

The RIL population was evaluated for seven different traits that included both morphological (PH, Rc, Ba), agronomic (SPLSPK, SpkLng, SPP), and domestication-related (Btr) phenotypes. Phenotyping was carried out for two consecutive years 2021 and 2022 (for details of phenotyping, refer to the Methods section). We observed genetic variability for all the traits with the coefficient of variation (CV) highest for spikes per plant (29.71 to 37.67) followed by spikelet number per spike, spike length, and plant height. Frequency distribution of the traits revealed normal distribution for all the traits as revealed by violin plots (Fig. 5; for Ba and Btr, refer to Supplementary Figs. 4 and 5, for Rc, refer to Fig. 6). Moreover, the correlation within the data for the two years revealed significant correlation for all the traits and highest correlation was observed for qualitative traits coleoptile color (0.50^***^), blue aleurone (0.98^**^) and for brittle rachis (0.82^***^). For the remaining traits, the correlation was lower at *r* = 0.40^***^ to 0.50^***^ but still highly significant (*P* < 0.001). However, the correlation of the individual year data with the pooled data again was high (>0.75^**^) (Fig. 5).

Both main effect and epistatic QTL were identified, and emphasis was also given to the pleiotropic QTL which showed QTL controlling more than one trait. The results are described in more detail as separate sub-headings below.

#### Main effect, epistatic and pleiotropic QTL

**Fig. 7:**
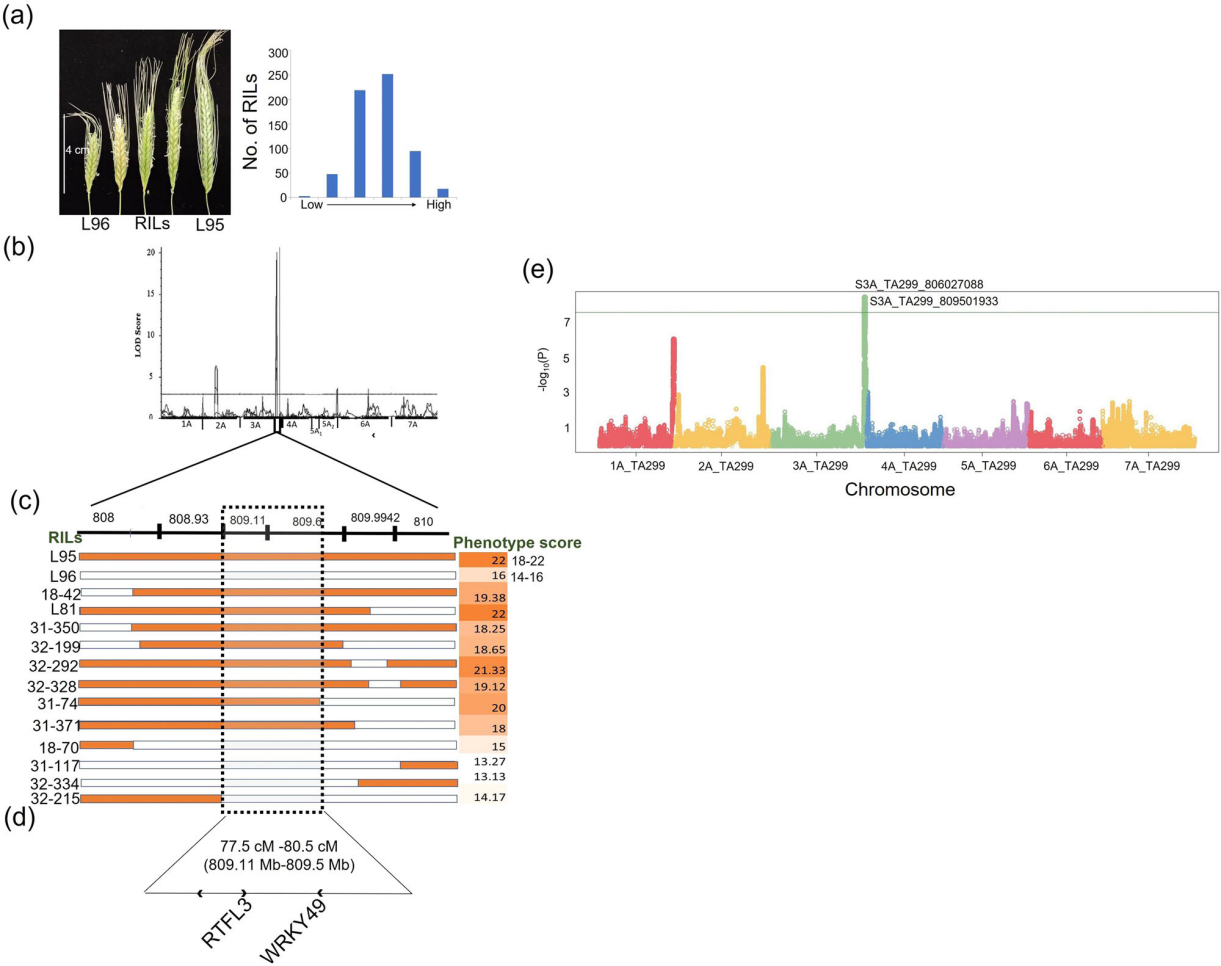
QTL and underlying candidate genes for spikelet number per spike. **a** Images showing the trait variation for spikelet number per spike and its frequency distribution depicted as histogram. The horizontal scale on the left side of the image showing trait variation for spikelet number per spike indicates the length of the smallest spike L96 (4 cm). **b** QTL peak obtained using IciMapping software (**c**) The text shown on the left of the horizontal bars indicate the codes for rRecombinant Inbred Lines (RILs) and the numbers shown on the right indicate the phenotypic trait values depicted as heatmap. L95 and L96 are the parental lines used for the generation of RILs. The horizontal bars indicate the regions covered by the QTL in the interval 808–810 Mb; each bar indicates region covered by each recombinant inbred line (RIL). The orange shaded regions in the horizontal bars represent the allelic regions for the parent 1 (i.e., TA4342-L95) whereas the white colored region in the horizontal bars indicate the allelic regions for the parent 2 (i.e., TA4342-L96). The highest trait values for the parental lines are mentioned in the heat map whereas the range is mentioned separately for both the parents (18 to 22 spikelets for L95 and 14 to 16 spikelets for L96). The range for spikelet number for both the parents is shown separately and the maximum value of the spikelet count for both the parents is depicted in the heat map. **d** The values mentioned in the brackets indicate the physical interval of the QTL region and the three candidate genes identified in the QTL interval region are also mentioned. **e** Manhattan plot for spikelet number per spike generated for MTA analysis using CMLM model.

A total of 36 main effect QTL and three epistatic QTL were identified for all the seven traits with a maximum number (11) of QTL for spike length and the least number (1) QTL for coleoptile color and blue aleurone respectively (Fig. 6; Supplementary Table 2; Supplementary Fig. 4). Out of the 36 QTL, four QTL were major QTL showing phenotypic variance explained (PVE) > 10 (Figs. 6 and 7; Supplementary Figs. 4 and 5; Supplementary Table 3) when verified using both QTL Icimapping ver 4.2 and R-QTL software (Supplementary Table 3), whereas the remaining QTL were minor effect QTL (Supplementary Table 3). The above QTL also included the QTL that were specific to one of the two environments or years (2021 and 2022) for quantitative traits (SPLSPK, SpkLng, PH, and SPP) which may be attributed to environmental variation. For qualitative traits (Ba and Rc), the QTL were common in both years which may be due to the high correlation observed within the environments (years) for these traits (>0.50**) (Supplementary Table 2). The two QTL for coleoptile color spanning a genetic distance of 21.5 cM to 23.5 cM (21.5 cM to 22.5 cM for 1st QTL and 22.5 cM to 23.5 cM for the 2nd QTL) were considered as a single QTL as the QTL intervals almost overlapped with each other.

A total of 3 important major epistatic interactions (Supplementary Table 4) were identified for the traits SPLSPK, SpkLng, and PH with the PVE 18.06%, 20.62 %, and 10.34%. The epistatic interactions involved chromosomes 1A and 3A. Furthermore, all three epistatic interactions showed negative additive by additive interactions.

An important QTL on chromosome 3A (*Q.SpnN/SpkLng/PH/SPP.3A*) was identified which was common for spikelet number per spike (SPLSPK), spike length (SpkLng), plant height (PH), and spikes per head (SPP). This included the region spanning from 79.5 to 80.5 cM (807 Mb to 810 Mb; Supplementary Fig. 6).

#### Fine mapping of spikelet number per spike (SPLSPK) and red coleoptile (Rc)

The available genomic (16.6 M imputed loci) and germplasm resources (sequence-indexed RIL population), allowed us to fine-map the major QTL for two traits which included one quantitative trait (spikelet number per spike or SPLSPK) and another qualitative trait (red coleoptile or Rc). The major QTL for SPLSPK (*Q.SPLSPK.3A)* was flanked by the markers at 808 and 810 Mb on chromosome 3A whereas the major QTL for Rc (*Q.Rc.umd.7A*) was flanked by the markers at 121 and 130 Mb.

For both the traits (SPLSPK and Rc), 12 and 13 recombinants were available in the RIL population, which helped us in further narrowing down the regions to 400 Kb for SPLSPK (809.11–809.5 Mb), and 700 Kb (124.3 to 125 Mb) for Rc (Figs. 6 and 7). Each of the two QTL regions harbored 4 (for Rc) and 3 (for SPLSPK) candidate genes in the interval. For Rc, an important candidate gene involved in anthocyanin regulatory protein was identified in the region along-with other candidate genes like Ubiquitin-like protein, zinc finger BED domain-containing protein, and putative AC9 transposase. Similarly, for SPLSPK, three candidate genes included transposon *TNT 1-94*, small polypeptide *ROTUNDIFOLIA LIKE 3*, and *WRKY49*.

### Marker trait association analysis

To assess the resolution of the combined high-density genetic markers obtained from skim-seq in the large RIL population, we conducted a standard MTA analysis. It is important to note that the MTA analysis was performed on the RIL population mentioned earlier to investigate the genotype-phenotype correlation in a segregating population, rather than on a diverse set of germplasm typically used for linkage disequilibrium (LD)-based GWAS. We have used the term "marker trait association (MTA)" throughout the manuscript to avoid confusion with GWAS analysis, which is commonly used for LD-based mapping analysis. It is worth mentioning that our study is not the first to utilize GWAS models for QTL identification in segregating RIL populations. Previous studies have explored the effectiveness of GWAS models in identifying QTL in crops such as cotton^46^ and soybean^47^.

Using MTA analysis with 1.6 million variants, we identified significant MTAs for almost all phenotyped traits. As we tested different algorithms, such as GAPIT and rrBLUP, for marker-trait association (MTA), we did not find any discrepancies in the association peaks. There was a strong concordance between the associations detected with the flanking regions of the identified QTL (Supplementary Table 5). This concordance is expected since the same populations were used for MTA analysis and linkage mapping. However, we also discovered additional loci through the MTA analysis that may be of potential interest and require further investigation (Supplementary Tables 2 and 5). Several loci were identified by both approaches. For example, for Btr using pooled data, we found three highly significant loci at 3AS, 4A, and 7A, which were also identified through interval mapping (Supplementary Tables 2 and 5). The MTA results for 4A and 7A overlapped with the genomic regions detected by the QTL analysis. Similarly, for coleoptile color, a strong hit (*P* value = 7.37E-19) was identified in the region at 123.5–124.6 Mb, which corresponded to the QTL region detected using interval mapping (Fig. 6 and Supplementary Table 5). Furthermore, for SPLSPK, three significantly associated loci were identified at 1A, 2A, and 3A. The locus identified in the telomeric region of the long arm of 3A at 808 Mb was the most significant, with several other significant loci between 806 and 810 Mb controlling SPLSPK. This finding also aligned with the QTL region identified using interval mapping (Fig. 7 and Supplementary Table 2). Similarly, the locus identified on 2A for SPLSPK corresponded to a QTL identified through interval mapping.

The MTA analysis also identified the unique regions associated with both domestication-related trait Btr and spike/spikelet morphology traits. Specifically, the locus identified on chromosome 3AS at 67–68 Mb for Btr was exclusively detected through MTA and not interval mapping. This locus was previously reported as a signature of einkorn domestication selection in earlier studies^48^. The locus for Ba on chromosome 4AL at approximately 432 Mb was also unique to MTA and differed from the locus identified on chromosome 4AS using interval mapping. Notably, the plant height loci at 1 A detected by MTA were not identified through interval mapping (Supplementary Table 5). Furthermore, the associations detected for SPLSPK and SpkLng at 1A and 5A, respectively, were exclusively found using MTA (Supplementary Table 5). These findings demonstrate that MTA provides enhanced resolution compared to linkage mapping and enables the identification of novel associations that may not be observed through interval mapping. Both approaches are complementary. Moreover, the precise genomic locations of the variants detected through MTA allowed us to conduct candidate gene searches within the same genomic region by aligning orthologs to the TA299 assembly.

### Candidate genes underlying associated MTAs

The important candidate regions identified solely through MTA analysis included the Btr1 loci at 3AS and the Ba loci at 4AL (Supplementary Table 5). These regions were further investigated through a candidate gene search (Supplementary Table 6). Nucleotide BLAST of the orthologs of known *Btr* genes (*Btr1-A, Btr2-A, Btr2-D-2)* from einkorn wheat, bread wheat, and *Ae*. *tauschii* (supplementary Note 3) against the TA299 reference assembly revealed the best alignments between 67 and 68 Mb on chromosome 3AS (Supplementary Table 6). These findings confirm that the MTA-detected Btr1 locus on 3AS is a significant region associated with the non-brittleness trait. Additionally, we attempted to map the orthologs of the blue aleurone gene (MYC4E) from *Thinopyrum intermedium* and *TbMYC4A* from the wheat-boeoticum substitution line^49^. However, no alignment was observed on the TA299 genome. Therefore, further examination is necessary to determine whether this region represents a novel locus contributing to Ba in einkorn wheat.

Further, the wheat gene for the coleoptile color anthocyanin regulation (*TaC1*-*B1*, LOC123157696) on chromosome 7B^50^ is also best aligned to the region on chromosome 7A in TA299 assembly where we identified a QTL through interval mapping as well as MTA analysis (124.6 Mb) for the trait Rc (Supplementary Table 5). This was one of the four genes for Rc identified using fine mapping (Fig. 6) and also showed polymorphism between the two accessions L95 and L96. Therefore, it is a potential candidate for Rc. This gene encodes for anthocyanin regulatory protein C1 and therefore is a potential target for future validation using transgenic approaches.

## Discussion

The present study in einkorn wheat was planned to develop a high-density bin map to validate newly constructed einkorn assemblies^30^ and then dissect key domestication and agronomic traits through interval mapping (using QTL IciMapping v4.2 software) and MTA analysis. Ahmed et al. (2023)^30^generated genome assemblies of a wild-einkorn accession TA299 and cultivated accession TA10622 and the genetic maps were useful in correcting the miss-orientation of scaffolds and validating the final assembly. Here we have extended the utility of this RIL population as a valuable genetic resource for precision trait mapping directly in einkorn wheat.

To translate the developed genomic resources for einkorn into agronomically useful information for breeding and wheat improvement, we explored the contrasting phenotypes for seven different traits (SPLSPK, SpkLng, SPP, PH, Ba, Rc and Btr) found in the RIL population. The parental accessions (L95 and L96) show contrasting phenotypes for several agronomically important traits including resistance against biotic and abiotic stresses. Therefore, the population developed using the above two accessions is an excellent resource for mapping several agronomically important genes^38^. The combination of large mapping population and high-density bin maps generated in the present study using affordable and low coverage skim-sequencing technology makes this resource an ideal choice to precisely map these traits and open opportunities to positionally clone the most important QTL or major genes.

In this study, we also showed that the skim-sequencing method^43^ is efficient in genotyping a larger population at a lower cost. With current sequencing output and library construction technologies, the genotyping cost for this skim-seq on large populations is in the range of 5–10 USD. The skim-seq was sufficient to delimit crossover breakpoints to intervals of 100–200 Kb from which we observed the recombination distribution followed a steeper U-shape pattern with the majority (91%) of the breakpoints in the distal 40% of the chromosome arms (Fig. 3). This doesn’t seem to be surprising due to the similar trend also observed in case of chromosome 3A in wheat^51^ and all the chromosomes in case of other gramineae family species like rice, maize^52^ as well as wheat ancestor *Ae*. *speltoides*^53^. Using the above resources, we further identified QTL for seven different traits and fine-mapped two traits including red coleoptile (Rc) and spikelet number per spike (SPLSPK). A novel domestication locus for brittle rachis was also identified on chromosome 7A (Figs. 6, 7, and S5).

Our results of the QTL for Rc largely coincide with the loci which were earlier identified on the short arm of group 7 (7AS) chromosomes in hexaploid wheat^54^ (*Rc-A1*, on 7A, *Rc-B1* on 7B and *Rc-D1* on 7D). The 7AS seems to be an important region of anthocyanin pigment genes as other loci related to anthocyanin pigmentation have also been mapped in the close vicinity of Rc genes which include the genes for purple culm (*Pc-A1*, *Pc-B1*, and *Pc-D1*), purple leaf sheaths (*Pls-A1*, *Pls-A2*, and *Pls-A3*) and purple leaf blades (*Plb-A1*, *Plb-B1*, and *Plb-D1*)^55,56^. The excellent germplasm and genomic resource also allowed us to fine-map the region to 700 Kb interval using the information on recombinants in the region. Further MTA analysis scan landed us on a candidate gene encoding for anthocyanin regulatory protein which is widely known to be involved in providing pink color to the coleoptile in *T. monococcum* population^57^.

Earlier, the QTL for Ba was mapped close to the centromere on the long arm of chromosome 4A^25,58^ and the closest marker reported by Dubkowsky et al. (1996)^58^ lies at ~491 Mb. This deviates from the location of the QTL for Ba identified in the present study (i.e., 4AS) using interval mapping which lies in the interval spanning 127 to 140 Mb. However, to some extent, it matches with the unique QTL identified using MTA analysis (at 432 Mb) which was not identified using interval mapping. The Ba trait is known to be controlled by either a single dominant gene *Ba1* derived from *Thinopyrum ponticum*^59,60^ or an incompletely dominant gene *Ba2* originating from *T. monococcum* or *T. boeticum*^25,58^. It would be interesting to plan a future study to examine the candidate gene for Ba based on the locus for this trait identified in the present study.

Pleiotropic QTL identified on chromosome 3A (*Q.SPLSPK/SpkLng/PH/SPP.3A;* Supplementary Fig. 6) should be an important region for further investigation for cloning of important gene(s) for yield-related traits and thereby targeted for marker assisted selection for yield improvement in wheat. An earlier report identified a similar QTL on chromosome 3AL using a RIL population with different accessions of wild and domesticated einkorn^36^. This QTL reported earlier controlled spikelet number per spike, spike length as well as earliness. Therefore, our locus on 3AL may correspond to the same locus identified in this earlier study. This region also harbors important candidate genes which are known to be involved in controlling the yield-related traits. Another report has identified a tillering mutant *tin3* (or tillering inhibition locus) that is mapped to this same region on the distal long arm of chromosome 3A^61^.

Further, three digenic (QTL x QTL) epistatic interactions were identified for the agronomic traits spikelet number per spike (SPLSPK), spike length (SpkLng), and plant height (PH) and all of them involved chromosomes 1A and 3A. While the markers with flanking the QTL on chromosome 1 were located 632 and 633 Mb, the markers for the second interacting QTL spanned the region between 808 and 810 Mb (for SPLSPK and SpkLng) which was the same region as our main effect QTL. The negative epistatic interactions for all three traits suggest that the epistatic effect was higher in the recombinants when compared to the parental genotypes. Further, no epistatic interactions for these traits have been previously reported in *T. monococcum* populations. These main effects and epistatic interactions identified for the agronomic traits may be helpful in the future for selecting an appropriate model for genomic prediction of these traits in *T. monococcum* as ignoring these effects in genomic prediction generally leads to low prediction accuracies^62,63^

Two major QTL (4A and 7A) and three minor effect QTL (1A, 2A, and 3A; Supplementary Fig. 5) were identified for brittle rachis in the present study. Ideally, if the distribution of the phenotype followed the expected mendelian segregation of 1:1, only a single major effect QTL may have been responsible for trait variation. However, in the present study, we also identified intermediate phenotypes for partially brittle rachis. The segregation ratio also deviated from the expected 1:1 ratio supporting an underlying genetic architecture of multiple QTL and or modifiers, leading to the observed mapping of multiple QTL for brittle rachis in the present study as also reported earlier^64^.

Our results of brittle rachis QTL also receive support from the earlier results where three QTL for *Btr* have been reported on chromosomes 3A, 4A, and 7A^32,64,65^. Two out of these three earlier reports^64,65^ reveal 3A to be a major QTL whereas the third report reveals QTL on 7A and 4A to be a major QTL in a population involving wild and domesticated einkorn accessions. The *Btr* locus on 3A in the present study was found to be homologous to the *Btr1* locus reported on chromosome 3H in barley^66^ and it is widely known to be major domestication locus that led to the divergence of wild and domesticated einkorn wheat. A similar locus (*Btr1*) was also reported by Adhikari et al. (2022)^48^ using a genome-wide F_ST_ scan in a *T. monococcum* panel consisting of 930 different accessions.

However, contrasting to the above, our results revealed the QTL on chromosomes 7A and 4A as the major effect QTL (Supplementary Table 2; Supplementary Fig. 5) which is not surprising as similar results were also reported earlier in a study involving an F_2_ population of wild and domesticated einkorn accessions^32^. A closer look at our results revealed 7A QTL to be more important (LOD of 21.18 and PVE of 13.28%); this locus was flanked by markers at 169 and 191 Mb; however, since the markers from 170 to 191 Mb were found to be co-segregating, the region between 169 and 170 Mb should be the important region for further fine mapping of the trait.

The *Btr* locus on 7A identified in the present study may harbor a novel Btr gene (*Btr3*) other than two barley homologs reported earlier in wheat (*Btr1* and *Btr2*)^64^ as we could not find these homologs in the region. Therefore, it would be interesting to explore the 7A locus identified here in more detail for its potential role in wheat domestication; future studies are being planned in this direction to validate this region. Overall, the QTL results of the Btr locus reveal a complex genetic model of spike brittleness as also shown earlier by ref. ^67^.

The QTL or the candidate genes for three of the traits, i.e., SPLSPK, Btr, and Rc showed colocalization with the QTL or candidate genes reported for these traits in earlier studies^32,36,68^. While the QTL for Btr and SPLSPK were earlier reported in *T. monococcum* itself, the candidate gene for Rc was reported in hexaploid wheat as well as in *T. monococcum*. We further examined the physical region for these earlier reported QTL to compare with the physical regions of the QTL reported in the present study. For SPLSPK, in the earlier study, three markers were reported closely linked to a pleiotropic QTL for heading time, anthesis time, spikelet number, and spike length and this region spanned ~5.1 Mb (806–811 Mb) region. This region contained 77 genes. The QTL for SPLSPK identified here was initially located in the 2 Mb interval spanning 808 to 810 Mb which we further narrowed down to a 500 Kb region containing three genes using the information on recombinants and 1.6 M SNPs (Supplementary Fig. 7).

For coleoptile color, the homoeologous region on group 7 chromosomes in bread wheat are widely known to contain genes for different pigmentation genes^54^ (*Rc-A1*, on 7 A, *Rc-B1* on 7B and *Rc-D1* on 7D). Himi and Taketa (2015)^68^ later identified candidate genes for Rc in the QTL interval on all the three sub-genomes known as *TaC1-A1*, *TaC1-B1*, and *TaC1-D1*. An earlier study in *T. monococcum* also identified a QTL on 7AS^25^ (Supplementary Fig. 8). In the present study, the gene for Rc identified on chromosome 7AS (at 124.6 Mb) showed similarity to the A genome homolog (*TaC1-A1*) identified in the above study^68^ This gene encodes for anthocyanin regulatory protein which is a kind of Myb transcription factor. In the case of *T. monococcum*, a homolog of this gene on chromosome 7AS was identified as a candidate gene based on the high expression observed in the cultivars with red coleoptile when compared to white coleoptile^57^. Similarly, in *Aegilops tauschii* RNA-seq analysis also revealed a similar protein encoding Myb transcription factor to be responsible for red coleoptile^69^. This gene was also found to be the homolog of the same gene identified on chromosome 7DS in hexaploid wheat.

For brittle rachis, a major QTL on chromosome 7AS was reported earlier in two different populations: one involving a wild einkorn accession ID228 (*T. monococcum* subsp. *aegilopoides*) and ID752, wild einkorn from Turkey (*T. monococcum* subsp. *aegilopoides*) and the second population involving ID396, a domesticated einkorn from Romania (*T. monococcum* subsp. *monococcum*) and the above-mentioned wild einkorn ID752^32^. The position of one of the two markers (left marker) flanking the QTL interval was at ~152 Mb whereas the position of the second marker could not be estimated as the sequence of the marker was not available. The 7AS QTL for brittle rachis identified in the present study spanned a distance of 21 Mb (from 169 to 191 Mb). Therefore, the earlier reported QTL most probably co-localizes with the QTL identified in the present study. Further analysis is being conducted to narrow down the QTL interval to identify a candidate gene at this novel Btr locus which has not been explored in detail to date. However, 3AS QTL is largely explored in wheat as well as in barley.

In this study, we demonstrated that low-coverage skim-seq genotypes with approximately 98% missing data could be imputed with an imputation rate exceeding 98% and an accuracy of approximately 95% (Fig. 5). The LB-Impute method proved to be highly valuable in imputing the missing data obtained from skim-sequencing. Additionally, the MTA analysis revealed several QTL (12 out of 17) that were also identified through interval mapping. However, the MTA analysis also detected unique regions that were not identified using interval mapping, which could be attributed to the large number of markers utilized in the MTA analysis. Notably, the MTA analysis identified a significant locus for Btr and Ba on chromosomes 3AS and 4AL, respectively, which provided further validation for previously reported QTL associated with these traits (Supplementary Tables 3 and 6). The overlapping genomic regions identified for traits like SPLSPK and SpkLng using both MTA analysis and interval mapping strongly support the observed positive correlation between SPLSPK and SpkLng (approximately 0.70** for pooled data). The genomic regions on 3A for SPLSPK and on 4A for Btr, among others, represent crucial regions controlling these traits (Supplementary Tables 3, 4, and 5). Therefore, the genomic regions identified by both methods can be prioritized for gene isolation and characterization.

In conclusion, the present study demonstrates the use of einkorn wheat as a diploid model to genetically dissect agronomically important traits, which can be more difficult in bread wheat due to its complex hexaploid nature and large genome size. Einkorn wheat, being the only diploid wheat having both domesticated and wild accessions, is proving to be an excellent resource for this purpose. Therefore, a large genetic mapping population using domesticated and wild-type accessions was developed for einkorn wheat and genotyped using a low-cost skim-seq approach.

When combined with the high-quality reference assemblies for einkorn, this large genetic population enabled precise identification of regions controlling domestication and agronomic traits on the genome. At least two traits including SPLSPK and Rc had a large effect QTL, from which we could fine map the trait-associated regions to small physical intervals (400 and 700 Kb), each containing only 3 to 4 genes. Interestingly, for Rc, we could even identify a putative candidate gene controlling anthocyanin pigmentation in the QTL interval. Thus, this study helps to show the great value and important place that einkorn has for functional genomics in Triticeae. The combined power of large populations and newly developed reference genome assemblies available for einkorn will further enable rapid advancements in gene discovery and characterization that will have direct applications in bread wheat and other important grain crops.

## Materials and methods

### Plant material

A RIL population consisting of 812 lines derived from a cross between a wild-type *Triticum monococcum* (*T. monococcum* spp. *aegilopoides*) accession TA291 (also identified as TA4342-L95) and cultivated *T. monococcum* (*T. monococcum* spp. *monococcum)* accession TA10868 (also identified as TA4342-L96) was used for construction of high-resolution chromosome bin-map. Out of the above 812 lines, a total of 635 lines could be phenotypically evaluated and therefore, used for the construction of the genetic linkage map and QTL analysis.

### Greenhouse experiments and phenotyping

For conducting the QTL analysis, the seeds of the above 635 lines were planted over 2 years (2021 and 2022) in 200 cell plug trays (each cell measures ¾” square by 21/4” deep; outer dimensions: 21¼” x 11¼” x 2¼” deep) with 5 seeds for each RIL. The soil used for initial planting was the SunGro propagation mix. The trays were initially kept in a mist chamber for germination for 5 to 6 days and then transferred to a vernalization chamber (4 °C, 12 h light, and 50% humidity) when the seedlings reached a 2-leaf stage.

After a vernalization treatment of 4 to 6 weeks, 2 (out of the 5) seedlings were transplanted to cones (height 1.5” and depth 8.25”) with 98 cones in each tray. The soil used for transplantation was a mixture of sungro LC1, sungro 360, and sungro green grave profile mix in the ratio of 2:1:1. The trays with the cones were allowed to grow in the greenhouse (23 °C-day, 18 °C-night, 12-h light, 50% humidity). Data were recorded for the seven traits mentioned earlier.

The detailed procedure followed for the phenotyping of the seven traits is explained below. The data was recorded in 2 replications over 2 consecutive years (2021 and 2022). Pooled data (the average of the data obtained in two individual years) was also used for interval mapping.

#### Coleoptile color (Rc)

The data for coleoptile color was recorded at 5 to 7 days after germination and the scoring was done as 1: for purple coleoptile 2 for intermediates and 3 for green color.

#### Blue aleurone (Ba)

The data for blue aleurone was also recorded as either 1 (for grains having an amber-colored aleurone layer) or 2 (for blue color). The scoring for blue and amber color was based on visual inspection of the grain aleurone color. Any grain showing a blue color (regardless of intensity), was scored as blue (score 2), and if amber color, scored as amber (score 1).

#### Spikelet Number Per Spike (SPLSPK), spike length (SpkLng), spikes per plant (SPP), and plant height (PH)

The data for SPLSPK and SpkLng were recorded on at least 3 mature spikes (including the main spike) and the average of the three mature spikes was considered for further analysis. The data for SPP and PH was recorded on 2 plants when the plants reached their physiological maturity.

#### Brittle rachis (Btr)

The data for brittle rachis was also recorded on three spikes per each replicate and the protocol adopted by ref. ^65^ was followed for estimating the brittleness of the rachis. Briefly, the mature spikes of plants from the RIL population and their parents were dried at 50 °C for 3 days. Subsequently, spikes with good seed fill were dropped from a height of 1.5 m. Spikes that disarticulated on impact were classified as brittle with a score of 3, and spikes that failed to disarticulate were classified as non-brittle with a score of 1. Spikes that were partially disarticulated were categorized as intermediates with a score of 2.

### Skim sequencing and bin mapping

The two parents of the RIL population (TA291 syn. TA4342-L95 and TA10868 syn. TA4342-L96) were sequenced at high depth (9.1x coverage) using a PCR-free Illumina TruSeq library while the RILs were sequenced in two sets using a low-volume Illumina Nextera library^43^. The first set consisting of 93 samples developed originally by ref. ^25^ with three blanks and was sequenced at 0.2x coverage and the second set consisting of 733 samples and was sequenced at 0.03x coverage with 35 blanks as control. Five replicates of each of the two RIL parents were also included along-with the RILs in the skim-seq panel.

Both the TruSeq and Nextera libraries were sequenced on Illumina NovaSeq with 2 × 150 bp reads (Psomagen Inc.). Demultiplexing of raw FASTQ files (https://github.com/sandeshsth/SkimSeq_Method) obtained from Nextera sequencing^43^ and TruSeq (https://github.com/sandeshsth/Fastq) was performed using custom perl scripts. Adapters and primers were trimmed using fastp70. Trimmed high-quality reads from the two parents were aligned to both the wild-type *T. monococcum* (*T. monococcum* subsp. *aegilopoides*) accession (TA299) genome and cultivated *T. monococcum* (*T. monococcum* subsp. monococcum) accession TA10622^30^ using SAMtools (v1.8) and variants were called using BCFtools (v1.9). However, the genetic analysis of traits we presented in this study was based on the SNPs called on TA299 (wild einkorn) assembly only. The variants were filtered for minimum and maximum filtered read depths of ≥6 and ≤100, respectively, and reference and alternate allele depths of ≥3. Missing and heterozygous genotype calls were removed. The same set of variants identified and selected for the WGS parent were called on the RILs using the skim-sequencing pipeline as described^43^. Because of low sequencing coverage, a bin mapping approach was used in 1-Mb sliding windows to call consensus genotypes^70^. Genotypes called on RILs were coded according to the parental SNPs replacing the genotypes as either wild (P1) or domesticated (P2). A consensus genotype was called within the 1-Mb sliding windows based on the proportions of P1 and P2 within the window. If P1/P2 ≥ 0.7, then the window was coded as P1, if P2/P1 ≥ 0.7, the window was coded as P2, otherwise as heterozygous (H). A custom python script was used to genotype the 1-Mb windows and identify the recombination breakpoints (https://github.com/laxmangene7/Skim-Seq-Population-Genotyping). The genotyping file with filtered recombination bins for missing and heterozygous loci and individual RILs were used to construct the genetic linkage mapping. A complete pipeline with the necessary bioinformatics workflow used to genotype skim-seq RILs and generate bin maps is available (https://github.com/laxmangene7/Skim-Seq-Population-Genotyping).

### Recombination bins

A sliding window pair was used to investigate each side of every position on the genome to identify where differing adjacent genotypes (…P1 - P1 - P1 - P2 - P2 - P2…) were observed. The windows utilized typed variant sites within a 6.5 Mb sliding window to identify if each position was a potential crossover site. A higher score for the region is achieved the greater the difference between the content of the two regions, which are put into one of four categories: P1, P2, Heterozygous if a region has close to a 1:1 marker ratio, or NA if the composition is outside of the previous thresholds. A recursive logic tree filters the highest scoring and most appropriate regions concerning the wide-scale surroundings. If no crossover was detected between two different regions, a second search will be performed with as large of a moving window width as possible based on the surrounding known recombination sites to ensure crossovers are detected in high sequence error-prone regions.

### Construction of linkage map

Since only 635 out of the total 812 RILs could be phenotyped as mentioned earlier, a separate linkage map was constructed only for QTL mapping. Genotyping data consisting of 5096 1-Mb binned markers was used for constructing the linkage map for 635 RILs. A linkage map was constructed using IciMapping version 4.2.53. The markers showing the segregation distortion (*P* < 0.001) and missing values (>20%) were removed from the analysis and the grouping was performed with LOD ≥ 3 and ordering was performed using SER (seriation) algorithm. Recombination frequencies were converted to centimorgan distance using the Kosambi mapping function. The marker density plot was generated for depicting the density of the markers on each chromosome using the R package CMplot.

### QTL analysis and fine mapping of two important traits

Initial QTL analysis was conducted using ICiMapping version 4.2.53 using the ICIM-ADD algorithm with 1000 permutations for identifying the significant QTL based on LOD values. Both the data for individual environments or years (2021 and 2022) and pooled data (average of the data for two years) were used for QTL analysis to identify the QTL specific to each environment and the common QTL in both environments. However, for fine mapping, the common major effect QTL identified for both the years and with the pooled data were used. The QTL showing PVE percentage >10 were considered major QTL and the remaining QTL were considered minor effect QTL. QTL showing epistatic interactions were also identified using the ICIM-Epi function. For this purpose, a linkage map constructed using 5096 bins (1 Mb) were used.

However, for further fine mapping of two important qualitative (Rc) and quantitative (SPLSPK) traits, the complete genotype data consisting of 16.6 M SNP variants generated through imputation (see below) and the available recombinants in the QTL intervals identified using linkage mapping were used. Using 16.6 M markers for constructing a linkage map is computationally demanding since most of the software for linkage map construction cannot use more than several thousand markers. Therefore, initial QTL analysis was performed using 5,096 bins and this QTL information and the 16.6 M markers were used to identify more recombinants in the QTL interval. This information finally enabled us to further delineate our QTL physical interval to less than 1 Mb regions (400 to 700 Kb).

### Skim-seq genotype imputation and marker trait association (MTA) analysis

The skim-seq genotyping data was imputed using LB-impute [https://github.com/dellaporta-laboratory/LB-Impute] algorithm^45^, which is specially designed to impute low-coverage data, in some cases, it can achieve above 99% accuracy at just 0.1x coverage. We ran LB-impute with its default parameters, except that we set the recombination distance parameter –recombdist to 1 Mb and set the parameter to resolve conflicts -resolveconflicts. We measured the accuracy by randomly masking 3 percent of the non-missing genotypes (in each RIL), and then checking the proportion of the genotypes we imputed correctly. The accuracy we report is the average accuracy over all RILs, i.e., the average proportion of correctly imputed genotypes over all RILs.

The full dataset of whole-genome markers consisted of 16.6 M imputed SNPs. This whole-genome dataset was used for MTA analysis as a complementary analysis to the interval trait mapping. As we had a RIL population with extremely high-density markers, several orders of magnitude larger than the effective number of recombinant bins, we used a random subset of 10% of all markers (1.6 M) to saturate the genome while running the MTA analysis in a computationally efficient manner. The random 10% subset was separated using the SelectVariants tool within GATK (https://gatk.broadinstitute.org/hc/en-us) software, which randomly selects a portion of variants from a VCF file. The MTAs were identified using GAPIT software and the Bayesian-information and Linkage-disequilibrium Iteratively Nested Keyway (BLINK) model^71^. The kinship matrix was used as covariates while running GAPIT for RILs. We used a false discovery rate as a method of correction for multiple testing issues. The BLINK has been considered as the more statistically powerful and computationally efficient method for GWAS. We also ran CMLM and rrBLUP to check the potential algorithm bias^72^. The rrBLUP method uses the additive relationship matrix (A matrix) to model the marker’s genetic effects using the A.mat function. The rrBLUP uses a mixed linear model for the association analysis. We identified MTAs for all the traits that we used for QTL interval mapping using the same phenotypic data used for the QTL mapping. The RIL’s phenotypes used for MTA were described in the aforementioned greenhouse experiment and phenotyping section. The candidate genes underlying the identified loci were searched in the TA299 genome either directly on the annotated file or by aligning the cloned candidate genes for the trait on the TA299 genome using ncbi BLAST (http://blast.ncbi.nlm.nih.gov/). For identifying the candidate region for *Btr* locus, the sequences of different reported *Btr* genes were used. The sequence of *Btr1-A* was from wild monococcum^73^, *BTR2-A* (LOC123057934) from bread wheat^50^, and *Btr2-D-2* sequence from *Aegilops tauschii*^74^ (Supplementary Note 2).

### Statistics and reproducibility

This study primarily focused on mapping traits of einkorn wheat using skim-sequencing of a larger recombinant inbred line (RIL) panel consisting of more than 600 lines. We mainly explored standard and established approaches for QTL (Quantitative trait loci) analysis and marker-trait association (MTA) analysis. The phenotyping of plants was conducted in the greenhouse on a single plant basis, and although a robust statistical analysis was not necessary, the phenotypic evaluation was carried out for two consecutive years. To combine the data from both years, we calculated the standard means for each RIL. For testing the reproducibility of the phenotype data collected over 2 years, pearson correlation coefficient was calculated using cor function in R and data was represented in the form of a corrplot.

For the imputation of the skim-seq data, we utilized LB-Impute method with default parameters. The imputation validation was performed as described earlier. Regarding marker-trait association, we employed standard regression models such as BLINK, CMLM and rrBLUP. These models allowed us to determine associated loci by using the phenotypic data and the skim-sequencing genotypic data.

For the identification of recombination breakpoints, please refer to the separate note (Supplementary Note 1) that describes the method used. The recombination breakpoints (Fig. 3), along with other graphs (Figs. 2 and 4), were plotted using the ggplot2 package in the R software. The required script for skim-seq data analysis and data visualization can be found at https://github.com/laxmangene7/Skim-Seq-Population-Genotyping/tree/main.

### Reporting summary

Further information on research design is available in the Nature Portfolio Reporting Summary linked to this article.

### Supplementary information

Supplementary Information

Description of Additional Supplementary Files

Supplementary Data 1

Reporting Summary

## Data Availability

The WGS parents (L95 and L96) demultiplexed data (fastq) and the raw sequence FastQ files of the skim-seq RILs along the separate index barcodes (i5 and i7) sequence files fastqs have been deposited at the National Center for Biotechnology Information (NCBI) SRA database with the BioProject accession PRJNA879879. The einkorn assemblies for TA299 and TA10622 are used to generate the genotyping information, and the RILs barcodes indices key file required for demultiplexing as well as the genotyping SNP matrix file used for the marker-trait association can be obtained at DRYAD [10.5061/dryad.v41ns1rxj] repository. The source data for the Figures is available in the GitHub repository (https://github.com/laxmangene7/Skim-Seq-Population-Genotyping/tree/main) in Supplementary Tables and/or Figures and any other remaining data are available from the corresponding author (or other sources, as applicable) on reasonable request.

## Code availability

All the statistical codes used in the present study can be accessed using the link https://github.com/laxmangene7/Skim-Seq-Population-Genotyping/tree/main.
