# Supplementary material for: Integration of genetic and genomics resources in einkorn wheat enables precision mapping of important traits
Source: Commun Biol. 2023 Aug 12;6:835. doi: 10.1038/s42003-023-05189-z (PMC10423216; doi:10.1038/s42003-023-05189-z)
Supplement: Supplementary file 1 — Supplementary Information [file 42003_2023_5189_MOESM1_ESM.pdf]

Supplementary Table 1. Details of a linkage map for QTL analysis

Chromosome	Total markers	Total genetic distance(cM)	Marker density (Marker/cM)
1A	164	89.59	1.89
2A	147	79.3	1.85
3A	168	85.3	1.97
4A	129	69.78	1.85
5A1	23	14.652	1.56
5A2	81	41.608	1.94
6A	157	114.67	1.37
7A	207	100.14	2.07
Total/average	1076	480.37	1.875

Supplementary Table 2 Details of QTLs identified for 7 different traits

Trait	Year	Chr	QTL	Confidence interval (CI)	QTL peak position	Flanking markers from the peak position	LOD(PVE)	Additive effect
Coleoptile color (Rc)	2021+2022	7A*	*QRc.umd-7A.1*	21.5-22.5	22	Chr7A_121 and Chr7A_123	32.18(38.32)	0.594
		7A	*QRc.umd-7A.2*	23.5-24.5	24	Chr7A_130 and Chr7A_131	11.20(9.59)	0.63
Blue aleurone (Ba)	2021+2022	4A	*QBa.umd-4A*	28.5-30.5	29	Chr4A_127 and Chr4A_140	19.89(13.45)	-0.178
Brittle rachis (Br)	2021+2022	7A	*QBtr.umd-7A*	27.5-28.5	28	Chr7A_169 and Chr7A_191	21.18(13.26)	-0.664
	2021+2022	4A	*QBtr.umd-4A.1*	46.5-48.5	47	Chr4A_625-Chr4A_626	13.43(7.41)	-0.474
	2021+2022	4A	*QBtr.umd-4A.2*	30.5-31.5	31	Chr4A_275-Chr4A_447	7.13(3.82)	-0.3452
	2021	1A	*QBtr.umd-1A.1*	56.5-57.5	57	Chr1A_562-Chr1A_563	3.03(1.62)	0.241
	2021	3A	*QBtr.umd-3A.1*	10.5-11.5	11	Chr3A_113-Chr3A-123	3.71(2.01)	-0.271
	2022	2A	*QBtr.umd-2A*	70.5-71.5	71	Chr2A_649-Chr2A_651	3.25(1.68)	-0.243
	2022	3A	*QBtr.umd-3A.2*	6.5-10.5	10	Chr3A_89-Chr3A_113	3.86(1.98)	-0.266
	2022	1A	*QBtr.umd-1A.2*	61.5-62.5	62	Chr1A_583-Chr1A_584	4.37(2.24)	0.28
Spikelet number per spike ( ) (SPLSPK)	2021+2022	3A	*QSplN.umd-3A*	77.5-78.5	78	Chr3A_808 and Chr3A_810	19.87(12.09)	-1.36
	2021+2022	6A	*QSplN.umd-6A.1*	38.5-41	40	Chr6A_730-Chr6A_731	3.55(1.81)	-0.503
	2022	2A	*QSplN.umd-2A*	25.5-31.5	28	Chr2A_39 and Chr2A_41	3.92(2.33)	0.59
	2021	6A	*QSplN.umd-6A.2*	65.5-66.5	66	Chr6A_78 and Chr6A_81	3.0(2.61)	0.68

Spike length (SpkLng)	2021+2022	3A	*QSkLng.umd-3A*	79.5-80.5	77	Chr3A_809 and Chr3A_808	20.43(8.39)	-0.34
	2021+2022	1A	*QSpkLng.umd-1A.1*	88.5-89.0	89	Chr1A_640 and Chr1A_643	15.94(7.31)	0.3056
	2021+2022	2A	*QSpkLng.umd-2A.1*	55.5-57.5	56	Chr2A_264 and Chr2A_358	12.71(5.19)	0.268
	2021+2022	7A	*QSpkLng.umd-7A.1*	14.5-15.5	15	Chr7A_83 and Chr7A_84	5.56(2.67)	-0.175
	2021+2022	4A	*QSpkLng.umd-4A.1*	17.5-19.5	19	Chr4A_17-Chr4A_18	9.81(3.85)	-0.23
	2021-2022	7A	*QSpkLng.umd-7A.2*	39.5-40.5	40	Chr7A_586-Chr7A_587	6.49(2.47)	-0.184
	2021+2022	4A	*QSpkLng.umd-4A.2*	44.5-46.5	46	Chr4A_623-Chr4A_625	6.07(2.32)	-0.179
	2022	1A	*QSpkLng.umd-1A.2*	87.5-89	88	Chr1A_637-Chr1A_638	18.7(7.02)	0.358
	2022	2A	*QSpkLng.umd-2A.2*	48.5-49.5	49	Chr2A_84-Chr2A_85	24.9(9.40)	0.414
	2021	1A	*QSpkLng.umd-1A.3*	84.5-85.5	85	Chr1A_632-Chr1A_633	6.27(3.51)	0.219
Spikes per plant (SPP)	2022	1A	*QSPP.umd-1A.1*	16.5-19.5	18	Chr1A_24-Chr1A_25	3.89(2.28)	-0.299
	2022	1A	*QSPP.umd-1A.2*	84.5-85.5	85	Chr1A_632-Chr1A_633	4.29(2.32)	0.30
	2022	3A	*QSPP.umd-3A.1*	46.5-47.5	47	Chr3A_742-Chr3A_744	3.10(1.65)	0.25
	2022	3A	*QSPP.umd-3A.2*	76.5-78.5	78	Chr3A_809-Chr3A_808	9.7(5.4)	-0.46
	2022	4A	*QSPP.umd-4A*	18.5-19.5	19	Chr4A_17-Chr4A_18	8.41(4.64)	0.42
	2021+2022	2A	*QSPP.umd-2A*	34.5-40.5	37	Chr2A_43-Chr2A_42	3.46(1.98)	0.29
	2021+2022	3A	*QSPP.umd-3A.2*	77.5-80.5	79	Chr3A_809-Chr3A_808	4.71(2.60)	-0.33
Plant height(PH)	2021	2A	*QPH.umd.2A*	62.5-63.5	63	Chr2A_577-Chr2A_586	7.97(6.57)	2.89
	2021	5A	*QPH.umd.5A*	0-0.5	0	Chr5A_2-Chr5A_5	3.67(2.90)	1.92
	2022	2A	*QPH.umd.2A*	23.5-26.5	25	Chr2A_30-Chr2A_37	4.04(3.29)	1.94
	2022	3A	*QPH.umd.3A*	78.5-80.5	80	Chr3A_808-Chr3A_810	10.94(8.92)	-318

*The two QTLs are considered as one as there is only a difference of 1cM in their genetic position.

Supplementary Table 3 Contribution of QTLs using Icimapping and RQTL

Trait	Chromosome	RQTL	ICImapping
		PVE (%)	
Spikelet number per spike	3A	16.84	11
Brittle rachis	4A	12.18	7
	7A	13.25	13.28
Coleoptile color	7A	38.32	28.33
Blue aleurone	4A	13.14	13.45

Supplementary Table 4 Details of epistatic QTLs for spike length, spikelet numbers per spike and plant height

Trait	Chr	P1 (cM)	Flanking markers	Chr	P2 (cM)	Flanking markers	PVE(%)	Add1	Add2	Add by Add
SpkLng	1A	85	Chr1A_632 and chr1A_633	3A	80	chr3A_808 and Chr3A_810	20.62	0.0399	-0.0.378	-0.226
SPLSPK	1A	85	Chr1A_632 and chr1A_633	3A	80	chr3A_808 and Chr3A_810	18.06	-0.0055	-1.08	-0.80
PH	1A	85	Chr1A_632 and chr1A_633	3A	70	Chr3A_794 and Chr3A_796	10.34	0.0128	-0.372	-2.012

Supplementary Table 5 GWAS identified trait-associated loci, their positions, candidate genes and the QTL near the loci. The trait abbreviations included Btr (Brittle rachis), Ba (blue aleurone), Rc (coleoptile color), SpkLng (spike length), SPLSPK (spikelet number per spike), and PH (plant height). Any loci identified when using one-year phenotype as trait value were tabulated with the respective year on the first column.

**Trait**	**Chr**	**Markers Position**	**P-values**	**QTL in the vicinity**
Btr*	3A	68632701	3.63E-12	
Btr (2021)	4A	353115733	1.70E-10	*QBtr.umd-4A.2*
Btr	4A	626518503	1.30E-08	*QBtr.umd-4A.1*
Btr	7A	165782974	3.72E-15	*QBtr.umd-7A*
Ba (2022)	4A	432358697	8.64E-10	
Rc#	7A	123575551	7.37E-97	*QRc.umd-7A.1*
SpkLng	1A	638950016	1.50E-09	*QSpkLng.umd-1A.1*
SpkLng	3A	807769884	2.34E-23	*QSpkLng.umd-3A*
SpkLng	7A	586040943	4.30E-09	*QSpkLng.umd-7A.2*
SpkLng (2022)	2A	261284022	6.64E-12	*QSpkLng.umd-2A.1*
SpkLng (2021)	4A	625744627	8.30E-11	*QSpkLng.umd-4A.2*
SpkLng (2021)	5A	614635344	5.10E-12	
SPLSPK	1A	638050398	4.58E-14	
SPLSPK	3A	808675740	2.76E-16	*QSplN.umd-3A*

SPLSPK (2022)	2A	38527399	7.71E-09	*QSplN.umd-2A*
PH	1A	642521462	4.77E-17	
PH (2022)	3A	807070618	1.05E-09	*QPH.umd.3A*

Chr = Chromosome; *the location of the MTA coincided with the known Btr genes (*Btr-1A, Btr-2A, Btr2-D-2*) on chromosome 3A; #the location of the MTA coincided with the known gene for coleoptile color *TaCl-B1* (Zhu et al. 2021)

Supplementary Table 6 Candidate genes alignment stats when aligned to the TA299 monococcum genome

Query Gene and source species	Alignment length (bp)	Aligned Chr	Identity %	Alignment Position Range	Bit Score	E-value
Btr1-A, wild einkorn	591	3A	100	68008322 - 68008912	1092	0.0
BTR2-A, bread wheat	675	3A	98	67971879 - 67972551	1173	0.0
Btr2-D-2, Ae. tauschii	597	3A	91	67971879 - 67972475	793	0.0
TaC1-B1, bread wheat*	913	7A	92	124647535 - 124648435	1277	0.0

Chr = Chromosome

* Only a portion of a query gene was aligned as the query sequence as the query sequence was 5 Kb long

Supplementary Notes

Supplementary Note 1 Recombination Breakpoint Detection

To determine the most accurate position of recombination breakpoints, both the macroscopic region layout-- its general allelic consensus, and obvious trends of regions at a chromosome-scale-- must be utilized to account for misidentified markers and thin data as well as the microscopic region-- marker to marker precision-- to ensure the precise location of the recombination break is identified between the two differing markers with as much resolution possible. Additionally, even at the F6-7 inbreeding generation, heterozygous regions should still constitute roughly 1-2% of the genome for any given RIL, making the ability for the algorithm to detect heterozygous sites crucial. Although there are previously described crossover detection methods (Huang et al. 2009), these lack the robustness to properly identify breakpoints in low marker density regions, identify heterozygous regions with precision, and maintain resolution in sequencing error-prone data. First, a roaming score was sequentially calculated along an entire chromosome: an allele belonging to parent 1 (L95), would be summed as +1 point. An allele belonging to the second parent, parent 2 (L96), would sum as -1 point (figure 1).

Each variant site with a neighboring allele of the alternate parents were identified as ‘bends’, and a moving window was used to analyze markers to the left and right of these positions. A dual window of a combined width (or diameter) of 6,500,000 bp was used instead of a fixed number of markers to account for varying marker density. After markers had been selected inside both windows, a simple linear fit using least squares regression was performed on each side to determine the slope of the line of best fit. If either paired window had too low of marker density as defined as less than 25% of the average marker density of the chromosome or constituting only 3 markers, it was skipped and marked as low confidence. If the slope of the line was within a given tolerance of +1, -1, or 0, and the collinearity of the points were above a threshold, then that side of the window would be designated as a P1, P2, or Heterozygous region respectively. A tolerance of ± 0.2 was allowed for a homozygous window, and a tolerance of ±0.4 was allowed for a heterozygous window. This leaves the ranges -0.8 to -0.4 and 0.4 to 0.8 unassigned.

Each window was given a score based on the difference between the two slopes of each side. If the moving window pair had differing regions on either side, it was considered as a Candidate Recombination Breakpoint (CRB). If the moving window had a score below 0.1 for the difference in slope between the two lines of best fit, then that position would be marked an ‘anchor’ site, as its sides represent very similar regions. If two bends were more than half the distance of the sliding window away from each other, that position was also tested and would result in an anchor site between them to ensure no region was unrepresented.

These CRBs and anchor sites (with their respective information) were arranged in a list by physical position. Sites were then filtered so that only the highest scoring CRBs and lowest scoring anchor sites exist within their local neighborhood (determined by the window radius of 2.25 Mb), so that only the highest-scoring candidates of their region-to-region transition remain. Anchor sites were merged if adjacent. Finally, using the anchor sites, the continuity between each site was tested-- that is, that the same type of region (P1, P2, or Heterozygous) would be matching from the right side of a CRB to the left of the next CRB continuously through the chromosome.

A recursive logic tree then filtered the CRBs down to the candidates that best represent the marker data seen. First, CRBs were removed if either of the two regions that were determined to be on either side of it could not match with any other CRB between two anchor sites. The highest scoring duplicate CRB between two anchor sites whose left and right regions matched the left and right anchor sites beside it was kept, the

duplicate being removed. After filtering, if there were no surviving CRBs between two differing anchor sites (there was not a recombination breakpoint found that fits the region transition required between the two anchor sites), a secondary macroscopic search algorithm begins. This approach is useful to finding a transition point between two differing regions in a wider scale. It first identifies the width of the uncertain region. The algorithm determines if it is searching for an upward or downward bend from the region types of the anchors. For example, a region transition from P1 to P2 would mean that the region between the +1 and -1 sloped anchor sites must bend downwards at some point. From between the closest two anchor sites, a sliding window of diameter n markers, with n equal to the marker distance between the two anchor sites, would investigate every bend of the data. Each score is recorded. The window with the highest score, and with the expected bend (upwards or downwards), would be inserted as a CRB between the two anchor sites. Upon reaching continuity through every anchor site and to each end of the chromosome, the CRBs were then considered high-confidence recombination breakpoints (RBs), and the recombination breakpoints of each chromosome under each RIL were recorded.

Supplementary Note 2

Linkage map for QTL mapping: The linkage map consisted of 1076 1-Mb binned markers distributed over all 7 chromosomes (8 linkage groups) with a total genetic distance of 480.37 cM (Supplementary Data 1; Supplementary Table 1). Chromosome 5A was split into 2 linkage groups with 81 and 23 markers in each linkage group. Maximum markers (201) were observed on chromosome 7A whereas the least number of markers (104) were observed on chromosome 5A (5A1 and 5A2). The highest number of markers per cM (marker density) was found on chromosome 7A (Supplementary Table 1; Supplementary Figure 3). Only 2 gaps of >10 cM were observed on chromosome 6A (linkage group 7) between the markers Chr6A_29 and Chr6A_30 (17 cM) and Chr6A_31 and Chr6A_32 (14 cM). Similarly, few small gaps were also observed on chromosome 1A and 3A in the telomeric ends. This may be attributed to the low density of markers at telomeric ends due to low mapping efficiency.

Supplementary Note 3 Brittle rachis (Btr2) gene sequences and sources

1. wild einkorn brittle rachis sequence (Btr1-A)

>MK439950.1 *Triticum monococcum* subsp. *aegilopoides* BTR1-A (Btr1-A) mRNA, complete cds

ATGGCGCAGCCACCGCAATGGAAGGCGATGTACCAGTATGTGGCGATACGGGCGCACGACG
GCTGCGCCCGCGTCGAGGAAAGTGTCGCCGCCGCGCGTAGGGAGCTGGCGTCCCCGCTGGT
GCTGGACACCCGCAACGCTGCTGGGAGTTACACCTTGTTGCATTCCGCGATGACCCACGTCG
AGCACGCGTCCGGCTGCCTCTCCGGCGTCATATTCAGCATGCTGGTGGCCGAGCTCCTGGCG
CTCCATGGCTGCGGGGCCGTCCCGTCGAGGCCGGTGGCTGGCATCGGTGACCTCCGCCGCGA
CCGCGACGACCACGACGAGTGGCTCGCTCTGAGCAGGCTCGAGGCCGCCAGGGAGCAGGCC
CAGGACGCGCTCCGCGGGGTGGAGGGTACCTTCACCCTCCTGGCCTCCGTCCGGTTCATGCT
TCACAGCCGGACCGCCGACGCTGCCGGGCGCCGGCAAGTCATGGAAGAGCAGCTCCACGCC
GCCGCCGTCGAACTCCAGGCCGTGGTGGGCAGCGTGGCCAACATGTCCGCGCTGGCCTTCTT
GGCCACCCAGCCTGCCATCCGCAACCGCATCCAGTGA

Ref: (Zhao *et al.*, 2019)

2. Wheat brittle rachis sequence Btr2 or BTR2-A (LOC123057934)

>NC_057800.1:c66204341-66203667

CCTCCTATTAACAGCGACACACACACACACACACACACACACAGAGAGAGAGAGAGAGA
GCCTACCGACGCGGGCAATGGAGCAGTGGAGGAGCACAGCGGCGGAGGCGTCGTCTCGCAG
CTTCACCAACATCAACGGGACGAATGCAATAGTCGACGCCATCACTGGCGCCCGCCAGCAG
TACCGCCTGGCCGCCGAGGACTGCCGCATGTTCCGCCCGGGCGTGCATCCCCTGCCCAACGC
CGGCCAGGGCGCTTCAGCAGGCGGCGACATCATCGACCTCGCCCTCGGCCGGATCAAGCGC
TTCAGCAGGTTCCACGCCGTAATGGGCAACGTCTTCTCCCTCTGCGCTGACCACATCGGGCT
CCAGGGCAACGCGCCATTGTGGCGCGACAGGTGGCAGCTCCACCACGCCGACGCTGCCCGC
CACGCGGAGACGGCGCTGCACTGCCTACACTCCGCCAAGTCGCACGGCCATGCGGCGCTCG
GCGTCTTCCACGTCATGCTCAGGCCGCCATCGCCGCGAGCGGTCGCCCACGCCTGGGCACCG
GCGGCCGAGCAGCTCCTGCGCGGCGCGATGGACGATCTGGCCATGGCGGAGGCCGCGGTGG
AGCGGATGCGCCCGGCCATTGTCGCCCAGTTCTTCGACGCCTCGATGCTTCTGCATGGCTGA

Ref: (Zhu *et al.*, 2021)

3. *Aegilops tasuchii* Btr2-D-2 (LOC109775647)

>NC_053037.2:c59500691-59500095 LOC109775647 [organism=*Aegilops tauschii]* [GeneID=109775647] [chromosome=3D]

ATGGAGCAGTGGAGGAACATGGCCGCGAAGGCGTCGGCTCGCAGCTTCACCTACATCAACG
AGACGAATGCAGTAGTCGAGGCCATCAATGGCGCCCGCCAGCAGTACCGCCTGGCCGCCGA
GGACTGTCGCAGATTCCGCCCGGGCGTGCATCCCCTGCCCAACACCGGCCAGGGCGCTTCAG
CAGGCGGCCTCATCATCGACCTCGCCATCGGCCGGATCAAGCGCATCAGCAGGTTCCACGCC
GTACTGGGCAACGTCTTCTCCCTCTGCGTTGCGCACATCGGTCTCCAAGCCAACACGCCGTG
CTGGTGGGACAGGTGGCAACTCCACCGCGCTGACGCTGCCCGCCACGCGGAGACGGCGCTG
CAGTGGCTACACTCCGCCAAGTCGCACGGCCATGCGGCCGTCGGCGTCTTCCACGTCATGCT
CAGGCCGCCATCGCCGCGAGCAGTCGCCTACGCCTGGGCACCCGCGGCAGAGCAGCTCCTG
CGCCGCGCGAATGACGATCTGGCCATGGCGGAGGCCGCGGTGGAGCGGATGCGACCGGCCA
TTGTGGCCCAGTACTCCGACGCATGTATGCTTCTGCATGGATGA

Ref: (Wang *et al.*, 2021)

4. Wheat anthocyanin regulatory gene (*TaC1-B1*)

>NC_057813.1:80033155-80038831 *Triticum aestivum* cultivar Chinese Spring chromosome 7B, IWGSC CS RefSeq v2.1, whole genome shotgun sequence

ATGCAGTGGGCGCGTGACGACTACCTCCGCGACGAGATGGTCCGGCAGCGCCGGACCCGGG
AGAAGATAGAAGTCCGCAAGCGTGGGCGCGAGGACAAGCTCGGCGTCGTGATCCTCGACAG
CGACGACGACGAGGACGCCCCCGGACCGTCCAACCCGCCGCGCCAACCGGGGGAGGGATGC
AGTATGGACGGCGGCGGTTGAGGCGGCGATGACGACGGCGGCGGCGGCGACTACACGCGGT
TCTACAGCCTCCTCGACATGTAGAACTGCAAGGGCGGGCGGCGGGGAGCGGCGAGGTAGAC
GGCGAGGAGCGGCGATGGAGACGGCGAGGGGCAGCCCCTAGTAGTTTTTTCTGTTTTTGTAA
AATATGTTTAAATTTGAACGAACTGGCCAATGTTTGCGTTAAATTTAAGCCGTGTTTGCGCCG
TATTCAACTTTTTCAAAAAAAAACGTGGGCGCCGCGACTCGGGGGCATCACGCCTCCAGCGC
GCCGGTTAGCGCCGGTGCGCCCCCAGGGGGCGATTTTTAGCCCCTCCTGGAGGGCCAACGGC
TGGAGATGCTCTAATTTTGCATTGGGAAACGCTATGTGATGGTAACATATTTATGTTACTTTA
TTTGCCTCTCTACTCATTAACTACTTGCCACCTCATCATTTTTGCTTATGTGACATCTATGTTA
CTCCTACTATGACCAGCCTTAGTTCCATAATTCTTGTCATGGTTTTAGTTTAATTCTGAACTA
AAAGCATGACAAGAATTATAAAACTGAGGGAATAGAAGTTAGCACATGACAACCAGTAATG
TTTGCCCTTTTAGCCACGCCGACCAAAACAGGGGAATCAAGTAATCGTTGGTATTTCTAAGC
ACTTTTTTTTTTCATTTAGAGTGTACGAATTTACATCTTCTAGCGCTTTGCTTTATGCATGACT
AGTGTTTGGTGGACCCTCTACATGATTTACTTCGGCTTTCTGATGGGATATGGTTTAATTTGC
TCATGTTTTCTTTCTAACAGGCTACATTTTGAACATATCAGAATTAAGGTATTTGGTTTCACA
TTTTTTTATTACCATAGAGTTCGAGAATGGACGGGGAATGTGTGCTGAGTAACGCTAGTCTT
CTGGGGAATGGAAAAACATGACATCGAGTGTGCTTTTGATTTCTTTTGTATATATAATTATTT
TGAAACCAAGTTTTTATAAGTAAGAAATAGACATACACACAGTTTTAAATAGTGTGCTATAA
CTTTTAAAGAACCCTCCCGCTAAAGCTATGCACCCTAAAGGATGTCTAATCAACCCTTGATTT
TTTCATGTCTGAACATGTGTATGTGCAGTATATGTGATATATCATATATGATGTCTGATTGTA

TGTTTTTCATTATTAGGCTATCACTATGTAATCGGTGCCTACTACTCCCCCGTCCCATAATGT
AAAACGTTTTTTGACATTAGAACGTCTTACATTATGGGACGGAGGGAGTACGTGAAAATGTT
GTCATCACATCAGCAGTGCCCTGAGAAAATATTGTGGCACCGCTTGTGTGTTTGCCGCATGC
GAAAGAGCAGGGCCCATGCATGCATAAAGTAGTTCAGGCAGCCAACTTTACTCCACACTAG
ATTTTTGCTATGGCATCGATTGGGCAGTGTAGGCCATCGCTGCCAAATGAGGTAAACTGATT
GGCCCGTTTTTAGCTTACACAGTTAAGGGCTTTCTTATTCTCGCGATTTAAGAAAAAAAAAC
GTTAAATAGTGCTTAGCTTCTATATCTTTTTGTAGCTTCTGTTATATATAACCGGCTATTTAGA
GCTTTAGTTATATATCATAAATTATTGGCTATCGAAGCCAATTAGAGCACCAATGATGATAA
GAGATAAGTCATTAACTGTGCGGTTTCTCGACGTTATTTAGAATCCTAAGTAGCATAGTTCA
AGGTATTTAATGGGACAATACAGCCAACAATTCCGCCTAAATCTTAACAAAGAGTCTCACTC
GATCTTAAAGAAGAGTCGATTGCATTTTCTACCTTCCACGTCAAAAATTATCCTACATAGTA
GACTATGAAGTTGGCGTGCGTATTTATAATATTGCCCTTACAATAAGAATGTGGCTAGGTCT
CAGTCGACTGAGATTTAACCAAGTCTCAATCAAGTGACATAACATCAAGAGAGA

CAAAGAAAAGAAACTGAAGATTTTTTTTTTGCACGGATCTTAATGTATGAGCTCGCGGATAT
AGCATCGACTGAGACTTAACGAAGTGAGACTTAGCAAGACTGTTACAATAATATTGCAATG
GAGGGAGTAATGTTTCCGCCACTTGGAGATAAAAGGCAACTATGAATAAGCCTCTGTTATAG
AAATATGACATCGAATTTGGACAGAAATCATCGCTGCTGCCAGCGATATATAACGGCTGATG
AACGGCCGTCCTGGTTACGTCCCTGTTCATGCACACATGGAAGCCCTCCCGGCCGTCTGGTC
CATCTAACACTTTGAGGAAAGTGCCGCTCCCGATGGCCGTGGATAGCCTTAAAGGTGCTAAG
CCGGCCACGTAGTCGCGCTGCGGCGCCGGTGTATAAATACCCCAACCACCCCTTCTGCCCGC
AGACCACTCCCCCTCTCTAACAAAGCACACACGGCGAGATAGAGATAGAGGGAGAGAGAGG
AGAGCGCTGCTATACACACGACAAGGATGGACGATTTACACACGATGCTACCCATCAAGAC
GTCCATTTACACAAGATGTACTGTATCAAGCCGTTCAATCAGCCATCGTGCATGGTTCGGCC
GGGCAGTGTCGTTCGCCCAGTAGTTTCGGAGAGAGGATGGGGAGGAGGGCGTGCTGTGCCA
AGGAAGGGGTGAAGCGAGGGGCGTGGACGAGGGACGCGGATTTTTCCTAACTAGTTAGGCG
CGCAACGCGTATCTAACCCACACATCCACCAGGAAGTCAAATCAATGAGGCCCACAAGCTG
TCAGCTTTGCATGGTATTACAACTACCAACGAATTTCGATAGTGCGTACGTGGAGATGTATT
CCACCTGATTGGACCGGCTGCGGGGCCAGCCCTATAGATTCGACTGTGGTCTACAGGTGCAC
ATGCATCCTCCCAGGGCCCTGCTCCCAGTACCACTTTTGTCCTTACCGATTAGTAGTACTGAT
CTAATAGCTGCCCATGCATGCACGAGCTCGCAGGATGAGTGAAGTGAGTGAACTCGATTAG
ATTCCTTAAATGTGCAGATTTTCTTCACTAGTATTAAATGAATTACTGTCTTGGTATAAAACT
CTGGTGCTAAAAAGTTCAGTTAAGTTGCGAACAAACATTCCGAGATAGGGAAACAAAAGTG
TTGCTGAAGAATTTCTGAGCCAACAAAAGTGTGGCTGAAGAATTTTTGAAGCTAAATTATTC
ATAGTTTTTGTATTAACATATTATGTAAATATACATTAATTTACTTAGTTGATTTTTTAATGGC
GAGCTCTTCAAAAAATAGTCCGCAAAATCCTTCATGCTTGTACACAAACGCACCGGGTATGC
TAGATTAAAGTTCATTTAAGTGGGCTTTCAGTAAAGTCCTTCGTGTTTGAACACAAAAGCAC
CGGGCATGCTATATTAAAATTTGTCATGCAGGGATCTATCCTTGTATAATCGCCCAATGAATT
ATGTAAAGTCCTTCATGTAGGTACACAAATGCACCGGGTATGCTAGATTAAAGTTTATTTAA
GTGGGGTCTATCTTTGTGTCCGGCGCTTTCAGTAAAGTCCTTCGTGTTTGAACACGAAAGCAC
CGGGCATGCTATATTAAAGTTTATCATGCGGGATCTATCCTTGTGCAATCGCCCAGTGAATT
ACGTAAAGTTCTTCATGCTTGTACAGGAACACACCGGGTATGCTGGATTAAAGCGCATTAAG
TGGGGGCCTATTTTTGTGCAATCGCACGGTCCTTTCAGTAAAGTCCTTCGTGTTTGATCACGA
AAGCACTGGGCATACTATATTCAAGTTTGTCATGCAGAAATCTATCCTTGTGCAGTCGCCCA
GTGAATTATGTAAAGTTCTTCATGTTTGTACACGAACGCACCGGGTATGCGAGATTAAAGTT
CATTGAGTGGGGTATATCTTTAAAGTGGGGTTGTACTTTTTCAAAAAAAGTTTCAGTTGACAT
TGAAATATTTTTCATGCATATGGTTGTTACCACGGTTATTTAAAAAAATAATCCACTCAAAAT

AAAGTTCTTCAATTTTTTCAATAATGGTCTTAAATGGCCAGAAAGAAAGTTTCATTTTCAGGC
CAAGATCTCGGCAGTCTCACGGCACAAAATCTCAGGGAAGAAGAAGCCCAGCCAGATCTAA
TCGGGTGTGTTTCAAATTGTGAAAGCTGGGAAGAGGAAGTGTGGTGTGTACTACTGCGTCGT
AGGTGTAGGTACTACTAGGTAGGAGCGTCAGGGGAGGCAGATACGATGTGACTGTATATTC
CTGTATAGCAAACCGGGTGTTGTTTATTTGGATTTTAAGGCTGCAACCAGTGGCCCACGGCG
CGCGGATCCATAGGAAGTAGCAGCGGCAGCGGTACGCGTTGCGCGCCGAACCGGTTAGTAA
TAGCCATATTCGCGTGGACGAGCAAGGAGGACGAAATCCTGGCTTCCTACGTCAAGGCCCAT
GGCGAAGGCAGGTGGAGGGAGCTCCCCCAGCGAGCTGGTACGTACTAATCCATCGTAATGA
CGTAATCCCTAGCTAGCCATATACACAGGACCACTCTGATTCTATCAATGGCGAGCTGGACG
GAGAGCGGGAGTTGACGCATGTGAACTGAATGAATCTGCATGCAGGTCTGCGGCGGTG

CGGCAAGAGCTGCCGGCTGCGGTGGCTGAACTACCTCCGGCCGAACATCAAGCGGGGCAAC
ATCTCCGACGACGAGGAGGAGCTCATCGTCAGGCTCCACGGCCTGCTCGGCAACAGGTGGT
CCATCATCGCCGGCAGGCTGCCCGGCCGAACAGACAACGAAATCAAGAACTACTGGAACAG
CACGCTCGGCAGGAAGGCGCTCCCCGCCCGCCCCGCCATTGCCGCGGCGAGGACCATCGCC
ACGCCCGTCGCCTCCGGCTCCTCCAGCTCCACCGCAGGAAACGCAGCTGTGGCGCTGTCCAC
CTCCGTCCCTGCTGTCCTCCACGCTGCGGCGCCTTCGTCGCCGACCGCCGCGGTGTGGGCGC
CCAAGCCCGTGAGGTGCACGGGCGGCCTCTTCTTCCGCCGGGAGACGCCGCCACCCGCGCCG
GTCGTCGCGGAGACGCGGGCCGGGGGAGAAGAGGGAGATGCCTGCAGCGGCAGCTGCTCG
GAGACGTCGTCGGCCGAGCCGTGCTCGTCGGGGTCGGGCGGGGGAGACTGGATGGACGACG
TGAGAGCCTTGGCCTCGTTCCTCGAGTCCGACGAGGAATGGCTCAAGTCCCTGCACATGGCC
GGTTAATTAACCTCTCACACGTGATCGATCCAGTTCAGTCACATGTACGTGGGTGCGCCGCT
GCACGTACGCTTTTAGAAAAGGAAACTGTATGTGTACTTGAACAGTGTGTATACGTACAAAT
GCACTTACGCCCGGGCGTATGTGACGGTACGTGGGTGGCTAGCTCCCGTACGTTACGACTGT
ACTGAGTTGTTCATATGTAAACGTGGTTTTGAGAAAAATACGTAAGCT

Ref: (Zhu *et al.*, 2021)

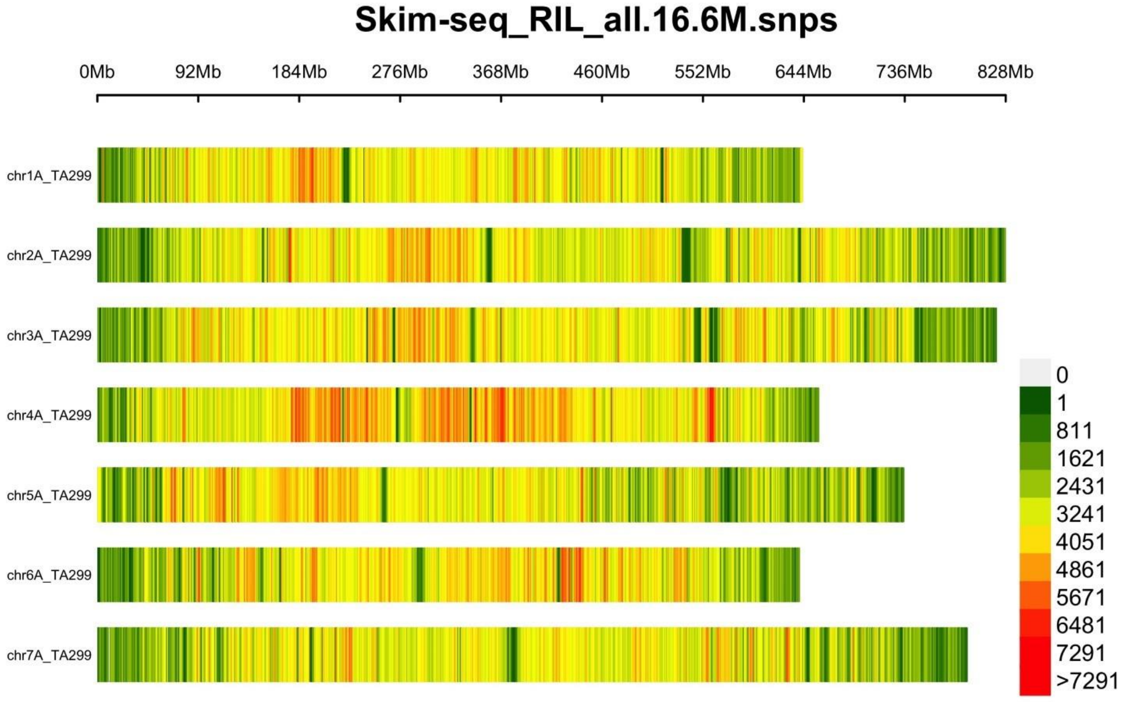

Supplementary Figure 1 Distribution of 16.6M variants in seven TA299 chromosomes.

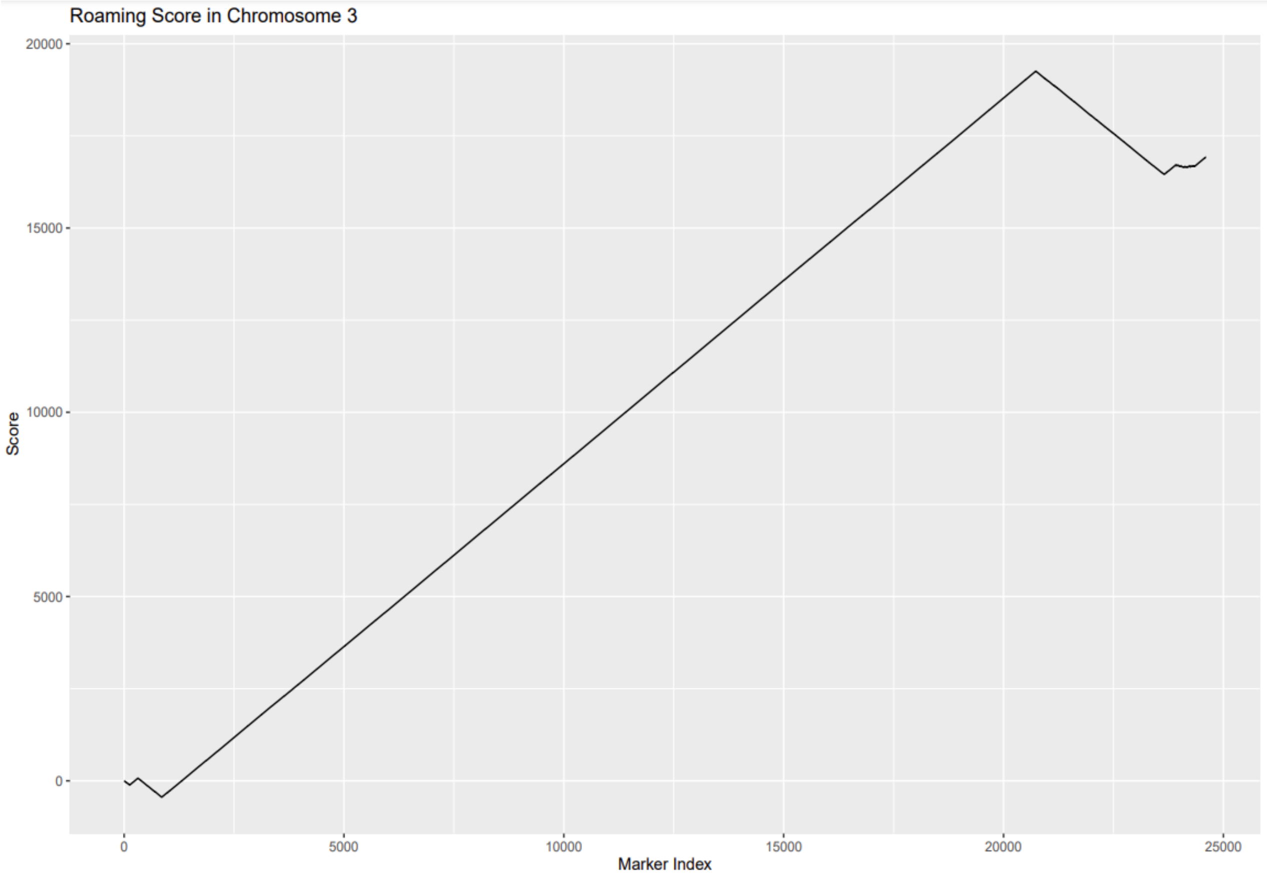

Supplementary Figure 2 Roaming score of Chr 3A on RIL 2013-18-107. X-axis representing the marker index of the variant, y-axis representing the roaming score based upon the parental allele. Positively sloped regions signify chromosomal regions belonging to P1, negatively sloped regions to P2, and flat or 0-sloped regions signifying a heterozygous region.

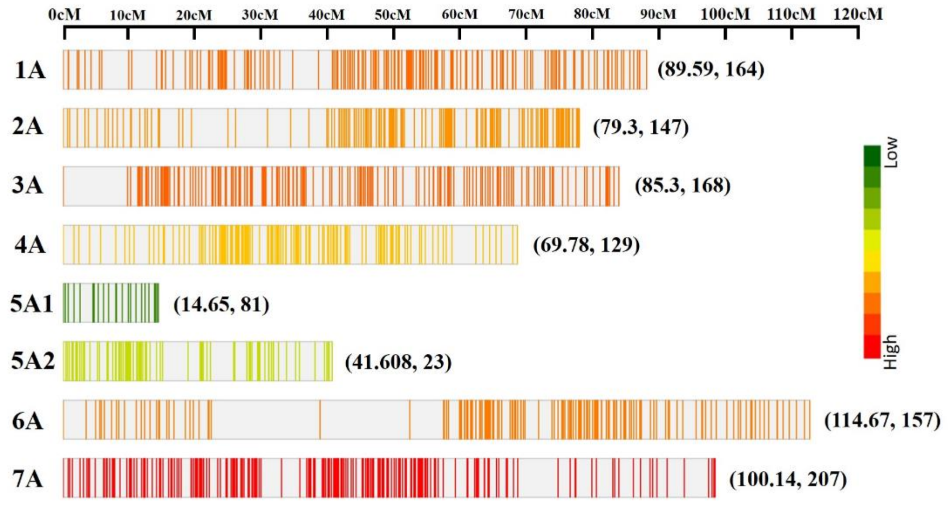

Supplementary Figure 3 1-Mb binned marker density plot for all the 8 linkage groups based on genetic(cM) distance . The figures in the parenthesis indicate the genetic distance in cM and the number of markers in each linkage group. X-axis indicates genetic distance in cM and Y axis indicates chromosome numbers.

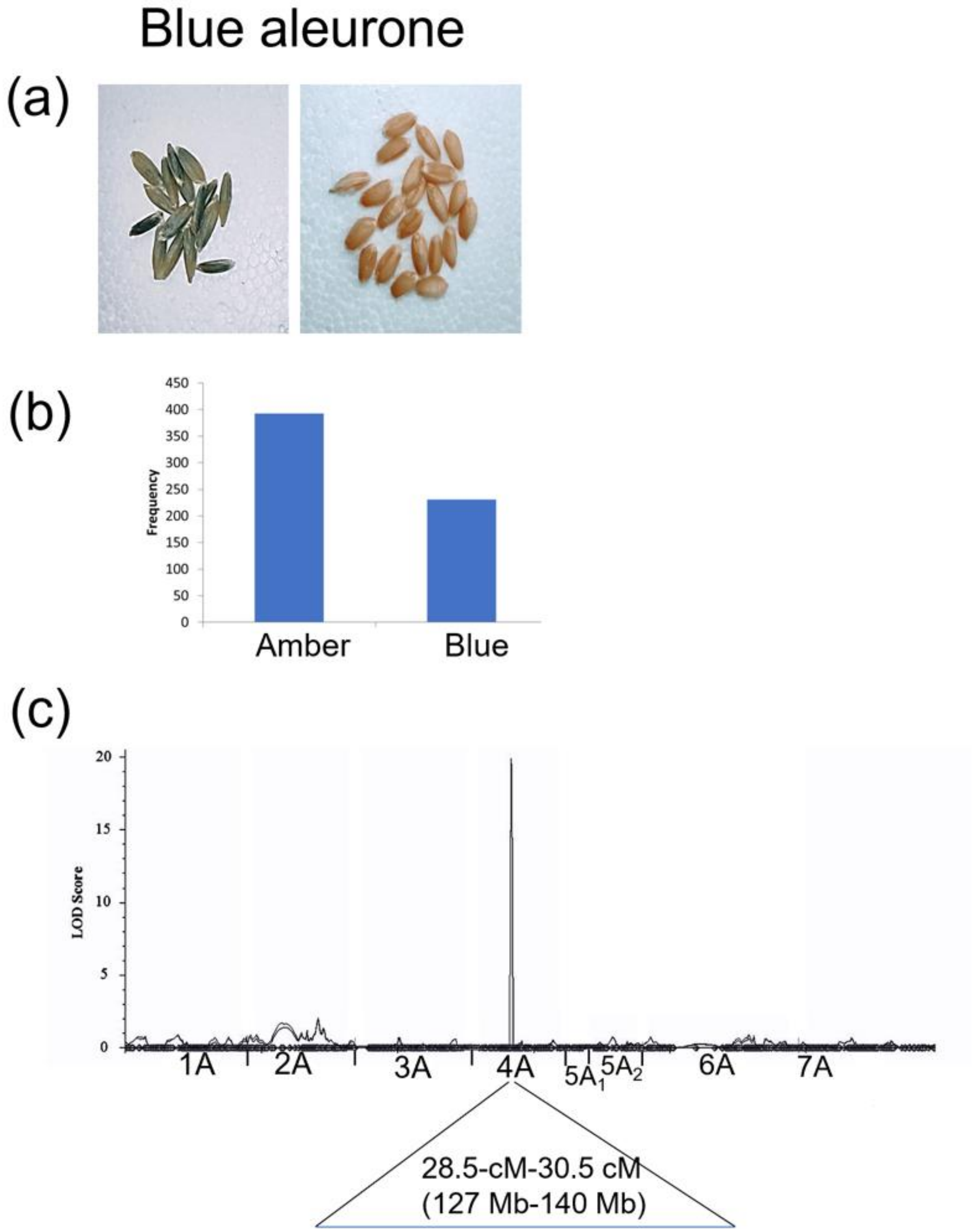

Supplementary Figure 4 QTL for blue aleurone (Ba); (a) L95 and L96 grains showing blue and amber aleurone; (b) histogram showing the distribution of Ba trait (c) QTL peak showing a major QTL on chromosome 4AS in the genetic interval 28.5 to 30.5 cM and physical interval 127 to 140 Mb.

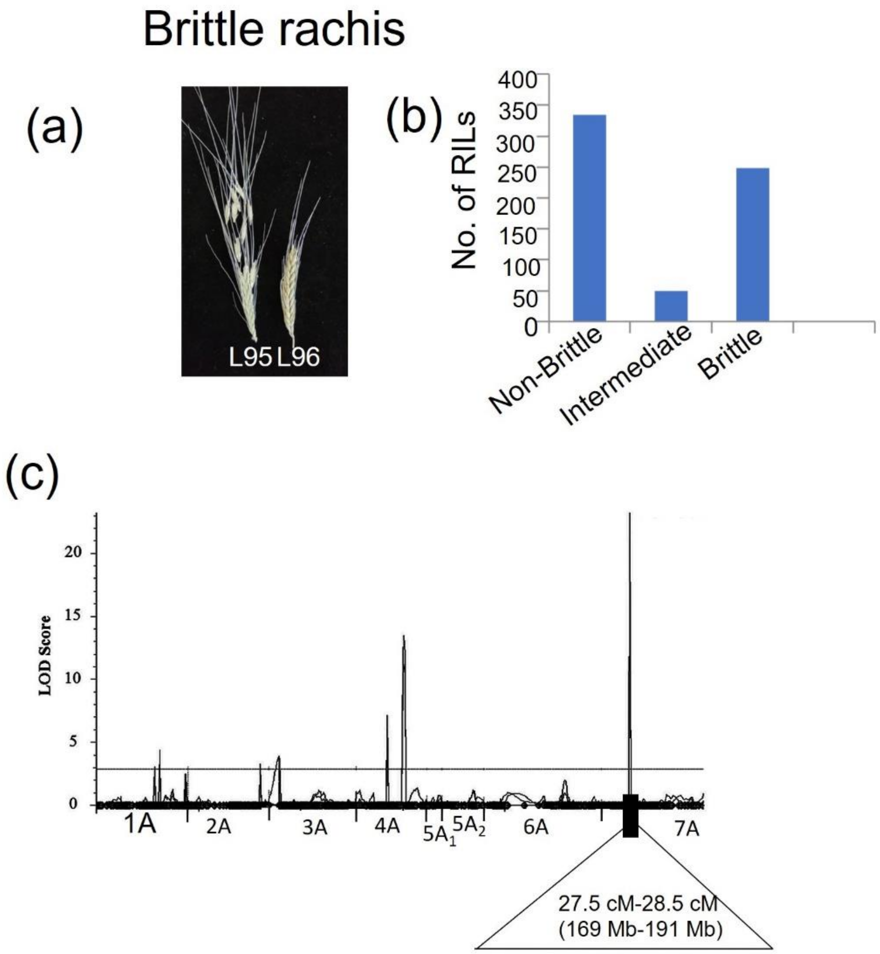

Supplementary Figure 5 QTL for brittle rachis (a) L95 and L96 grains showing brittle (Btr) and non-brittle spikes; (b) histogram showing the distribution of Btr trait (c) QTL peak showing a major QTL on chromosome 7AS in the genetic interval 27.5 to 28.5 cM and physical interval 169 to 191 Mb.

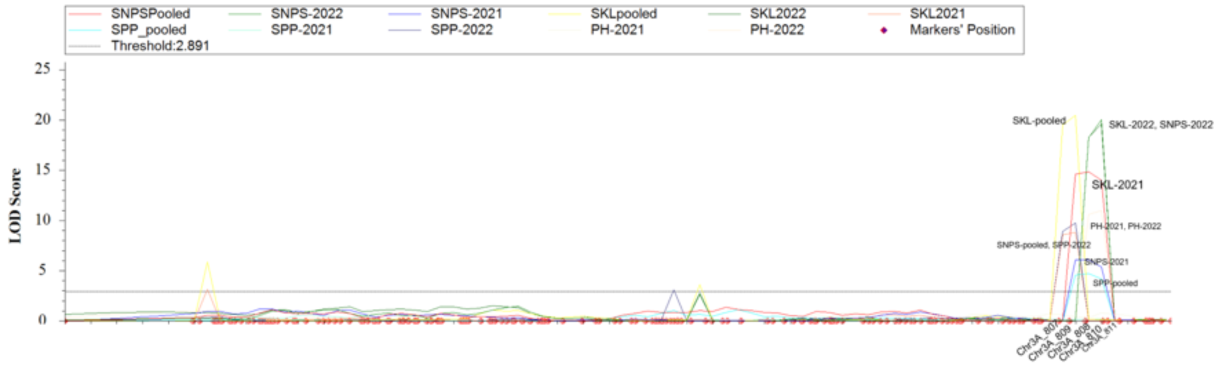

Supplementary Figure 6 A chromosomal map for chromosome 3A showing the common QTL identified in the interval between Chr 3A bins at 807 Mb to 810 Mb for the three yield related traits including spikelet number per spike (SPLSPK), spike length (SL), spikes per plant (SPP) and plant height (PH). The QTL was identified for either of the two years or both the years (2021 and 2022). Only some of the representative markers were used for preparing the map figure.

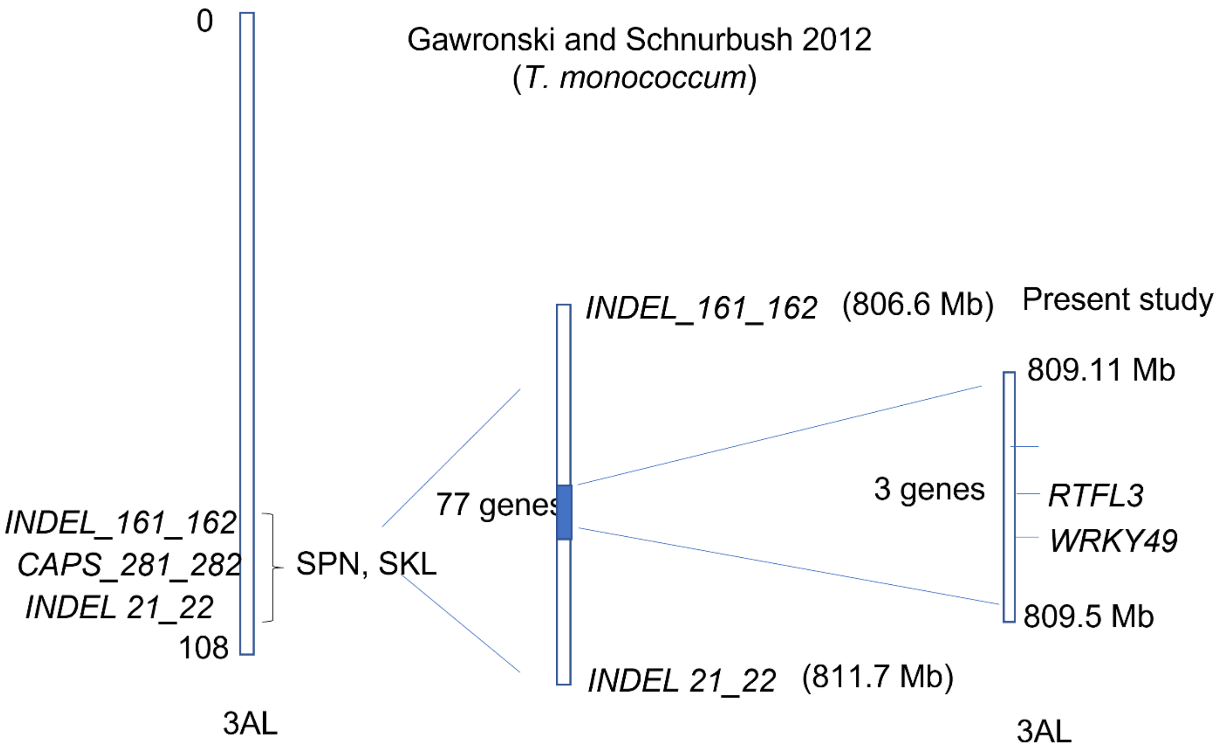

Supplementary Figure 7 Comparative analysis of a QTL for spikelet number per spike reported earlier in *T. monococcum* (Gawronski and Schnurbush 2012) and in the present study on chromosome 3AL.

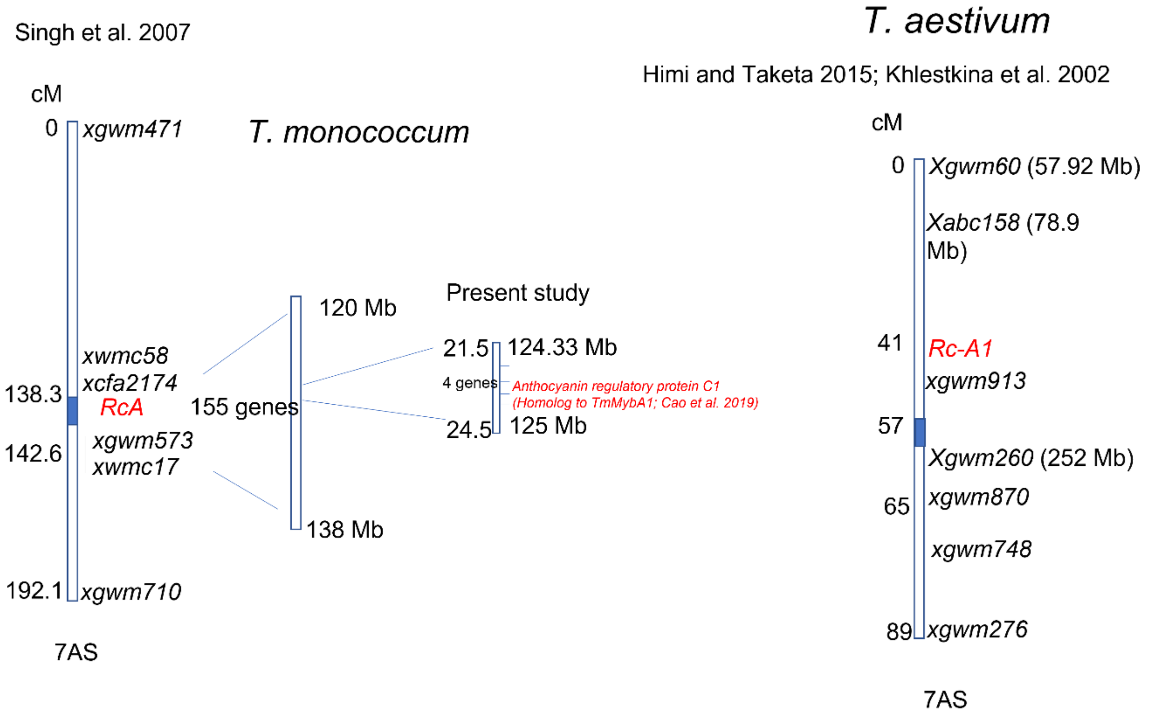

Supplementary Figure 8 Comparative analysis of a QTL for coleoptile color reported earlier in *T. monococcum* (Singh et al. 2007) and in the present study along with a QTL reported earlier in hexaploid wheat on chromosome 7AS (Khlestkina et al. 2002; Hima and Takita 2015).

Supplementary References

1. Wang L, Zhu T, Rodriguez JC, Deal KR, Dubcovsky J, McGuire PE, Lux T, Spannagl M, Mayer KFX, Baldrich P, et al. 2021. Aegilops tauschii genome assembly Aet v5.0 features greater sequence contiguity and improved annotation. *G3 (Bethesda, Md.)* 11(12).
2. Zhao Y, Xie P, Guan P, Wang Y, Li Y, Yu K, Xin M, Hu Z, Yao Y, Ni Z, et al. 2019. Btr1-A Induces Grain Shattering and Affects Spike Morphology and Yield-Related Traits in Wheat. *Plant and Cell Physiology* 60(6): 1342-1353.
3. Zhu T, Wang L, Rimbert H, Rodriguez JC, Deal KR, De Oliveira R, Choulet F, Keeble-Gagnère G, Tibbits J, Rogers J, et al. 2021. Optical maps refine the bread wheat Triticum aestivum cv. Chinese Spring genome assembly. *The Plant Journal* 107(1): 303-314.
