# Supplementary material for: Integration of genetic and genomics resources in einkorn wheat enables precision mapping of important traits
Source: Commun Biol. 2023 Aug 12;6:835. doi: 10.1038/s42003-023-05189-z (PMC10423216; doi:10.1038/s42003-023-05189-z)
Supplement: Supplementary file 2 — Description of Additional Supplementary Files [file 42003_2023_5189_MOESM2_ESM.pdf]

## Description of Additional Supplementary Files

**File name:** Supplementary Data 1
**Description:** Genetic linkage map
